# Vitamin D and Ocular Diseases: A Systematic Review

**DOI:** 10.3390/ijms23084226

**Published:** 2022-04-11

**Authors:** Hei-Nga Chan, Xiu-Juan Zhang, Xiang-Tian Ling, Christine Huyen-Trang Bui, Yu-Meng Wang, Patrick Ip, Wai-Kit Chu, Li-Jia Chen, Clement C. Tham, Jason C. Yam, Chi-Pui Pang

**Affiliations:** 1 Department of Ophthalmology and Visual Sciences, The Chinese University of Hong Kong, Hong Kong, China; hnrubychan@cuhk.edu.hk (H.-N.C.); zhangxiujuan@cuhk.edu.hk (X.-J.Z.); lingxt@link.cuhk.edu.hk (X.-T.L.); huyentrangbui@cuhk.edu.hk (C.H.-T.B.); yumengwang@cuhk.edu.hk (Y.-M.W.); waikit@cuhk.edu.hk (W.-K.C.); lijia_chen@cuhk.edu.hk (L.-J.C.); clemtham@cuhk.edu.hk (C.C.T.); 2 Department of Paediatrics and Adolescent Medicine, University of Hong Kong, Hong Kong, China; patricip@hku.hk; 3 Hong Kong Hub of Paediatric Excellence, The Chinese University of Hong Kong, Hong Kong, China; 4 Department of Ophthalmology and Visual Sciences, Prince of Wales Hospital, Hong Kong, China; 5 Department of Ophthalmology, Hong Kong Children’s Hospital, Hong Kong, China; 6 Hong Kong Eye Hospital, Hong Kong, China

**Keywords:** vitamin D, ocular disease, vitamin D receptor, myopia, age-related macular degeneration, glaucoma, dry eye syndrome, thyroid eye disease, uveitis, retinoblastoma

## Abstract

The contributory roles of vitamin D in ocular and visual health have long been discussed, with numerous studies pointing to the adverse effects of vitamin D deficiency. In this paper, we provide a systematic review of recent findings on the association between vitamin D and different ocular diseases, including myopia, age-related macular degeneration (AMD), glaucoma, diabetic retinopathy (DR), dry eye syndrome (DES), thyroid eye disease (TED), uveitis, retinoblastoma (RB), cataract, and others, from epidemiological, clinical and basic studies, and briefly discuss vitamin D metabolism in the eye. We searched two research databases for articles examining the association between vitamin D deficiency and different ocular diseases. One hundred and sixty-two studies were found. There is evidence on the association between vitamin D and myopia, AMD, DR, and DES. Overall, 17 out of 27 studies reported an association between vitamin D and AMD, while 48 out of 54 studies reported that vitamin D was associated with DR, and 25 out of 27 studies reported an association between vitamin D and DES. However, the available evidence for the association with other ocular diseases, such as glaucoma, TED, and RB, remains limited.

## 1. Introduction

Vitamin D has diverse functions in maintaining human health, including regulating gene expression, immune system, inflammation, cell proliferation and differentiation, apoptosis, and angiogenesis [1,2]. Vitamin D_3_, or cholecalciferol, is produced from its precursor, 7-dehydrocholesterol, in the epidermal layer of skin under exposure to sunlight, or is obtained from the diet. It is metabolized in the liver and kidneys to its biologically active forms, 25-hydroxyvitamin D (25(OH)D_3_) and 1,25-dihydroxyvitamin D (1,25(OH)_2_D_3_), respectively. The latter is also known as potent steroid hormone calcitriol. Reduced sun exposure will lead to vitamin D deficiency [3,4]. Low vitamin D levels have been associated with many diseases, including cardiovascular diseases [5,6], hypertension [7], diabetes mellitus [8,9], and cancers [10]. 

The vitamin D status of an individual is usually determined by serum 25(OH)D_3_ instead of 1,25(OH)_2_D_3_ because of its longer circulating half-life and higher concentration in circulation [11]. Besides, 1,25(OH)_2_D_3_ levels are affected by calcium levels [12,13]. Even though a range of thresholds is used between various scientific societies, having blood levels lower than 12 ng/mL of 25(OH)D_3_ represents deficiency, 12–20 ng/mL represents insufficiency, 20–100 ng/mL represents sufficiency, and >100 ng/mL indicates a risk of toxicity [14].

## 2. Metabolism of Vitamin D

**Figure 1:**
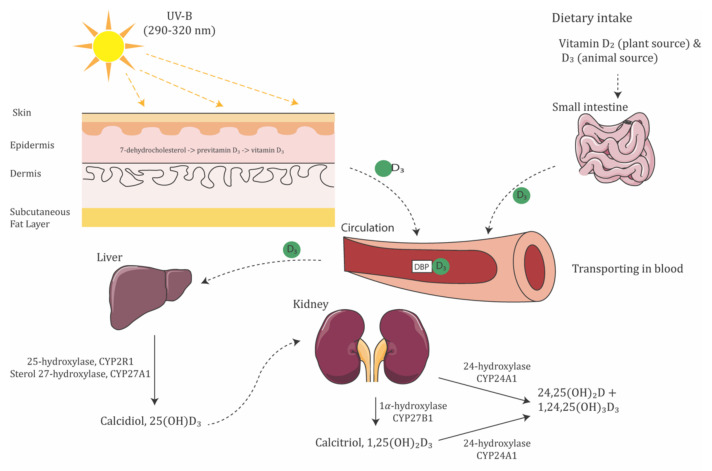
Schematic illustration of vitamin D synthesis pathway. Moreover, 7-dehydrocholesterol in the epidermis layer of skin absorbs UV-B radiation and is converted to pre-vitamin D_3_. Vitamin D_3_, by either the isomerization of pre-vitamin D_3_ in the epidermal basal layers or intestinal absorption from the diet, binds to vitamin D-binding protein (DBP) in the bloodstream, transported to the liver. Vitamin D_3_ is hydroxylated by 25-hydroxylase or sterol 27-hydroxylase. The resultant calcidiol (25(OH)D_3_) is 1α-hydroxylated in the kidney by 1α-hydroxylase, yielding biologically active vitamin D (1,25(OH)_2_D_3_).

Vitamin D is synthesized and activated in three steps (Figure 1). Cholecalciferol (vitamin D3) and ergocalciferol (vitamin D2) are the two major biologically inert precursors of vitamin D. For the former, 7-dehydrocholesterol in the skin produces previtamin D3 under exposure to ultraviolet B radiation (UVB, λ = 290–315 nm), which then thermally isomerizes to Vitamin D_3_ in the skin; in contrast, vitamin D_2_ is derived from plants and obtained from the diet. After its production, vitamin D_3_ attaches to vitamin D-binding protein (DBP) in the liver, where it is activated to produce 25(OH)D_3_, the primary circulating form of vitamin D, by 25-hydroxylases, CYP2R1 and CYP27A1. Then, 25(OH)D_3_ is converted to 1,25(OH)_2_D_3_, the active form of vitamin D, by 1α-hydroxylase, CYP27B1. In contrast, vitamin D_2_ is derived from plants and obtained from the diet. CYP27A1 does not hydroxylate vitamin D_2_ at the 25 positions. Lastly, vitamin D metabolite levels are downregulated by CYP24A1, which catalyzes the 24-hydroxylation of both 25(OH)D_3_ and 1,25(OH)_2_D_3_ [15]. The genetic variation in the metabolic enzyme would affect the regulation of vitamin D levels. 

Moreover, 1,25(OH)_2_D_3_ can penetrate the cell membranes, either as a free molecule or in DBP-1,25(OH)_2_D_3_ complexes. It then binds to the vitamin D receptor (VDR), facilitating the interaction of VDR with the retinoic X receptor (RXR) [16]. This VDR-RXR heterodimer binds to both positive and negative vitamin D response elements in target genes, influencing gene transcription [17]. Hence, the presence of VDR suggests the local activity of vitamin D [18]. In particular, VDR has been detected in different parts of the eye, including the epithelium and endothelium of the cornea, lens, ciliary body, retinal ganglion cells (RGCs), inner nuclear layer, photoreceptors, and retinal pigment epithelium (RPE) [19,20]. Genetic alternations of the VDR gene could lead to defects in gene function, calcium metabolism, cell proliferation, and immune function. DBP is mainly responsible for the transportation of vitamin D and its metabolites.

Levels of active vitamin D in the body are regulated by the enzymes 25-hydroxylase, 1α-hydroxylase, and 24-hydroxylase [15]. In a recent study, the 25(OH)D_3_ and 1,25(OH)_2_D_3_ generating enzymes 25-hydroxylase (CYP2R1 and CYP27A1) and 1α-hydroxylase (CYP27B1), as well as the deactivating enzyme 24-hydroxylase (CYP24A1), were found to be strongly localized at the complementary regions of the ciliary body, RPE, neural retina, corneal epithelium and endothelium, and scleral fibroblast, suggesting that vitamin D in the eye is locally produced, activated, and regulated [21,22]. Moreover, vitamin D-dependent calcium binding protein calbindin, a vitamin D metabolizing protein, was shown to be expressed throughout the human retina [23]. Some of the cohort studies reported the correlation of metabolic enzymes in ocular diseases. In diabetic patients, retinal CYP27B1 was found to correlate strongly with VEGF-A in the eyes [24]. In a cohort of patients with Vogt-Koyanagi-Harada disease, a non-synonymous variant of CYP2R1 was found in 17 of 39 patients, suggesting that the variant in CYP2R1 may play a role in VKH pathogenesis [25].

Some of the vitamin D regulating proteins, such as ferredoxin reductase participating in the activation of vitamin D in the kidney, are metalloproteins. Vitamin D is able to interact with the matrix metalloproteinase. Metal deficiency may affect ocular condition. However, only one study found significantly lower serum calcium levels in blepharospasm patients, but no significant difference in magnesium, phosphorus, or vitamin D [26]. 

Therefore, the potential of vitamin D to regulate various processes of potential relevance to ocular diseases has been acknowledged. Studies investigating the roles of vitamin D in ocular tissues and ocular disease pathogenic pathways have been carried out and will continue to contribute towards our understanding of ocular disease mechanisms and help establish effective intervention.

## 3. Vitamin D and Ocular Diseases

The potential effect of vitamin D deficiency on human health is a big concern. Recently, especially over the past few years, since the last published review articles related to vitamin D and ocular diseases, more and more studies investigating the relationship between serum vitamin D level and ocular diseases were published, including some prospective studies examining this relationship and therapeutic effects of vitamin D. Currently, review articles related to vitamin D and ocular disease are available [18,20]. To update this and reach a comprehensive understanding, hence, we performed a systematic review here to summarize the evidence revealing the association between vitamin D and ocular diseases.

### 3.1. Method of Literature Search

This systematic review was conducted in accordance with the Preferred Reporting Items for Systematic Reviews and Meta-Analyses guidelines [27]. The protocol is described as follows.

#### 3.1.1. Search Strategy

**Figure 2:**
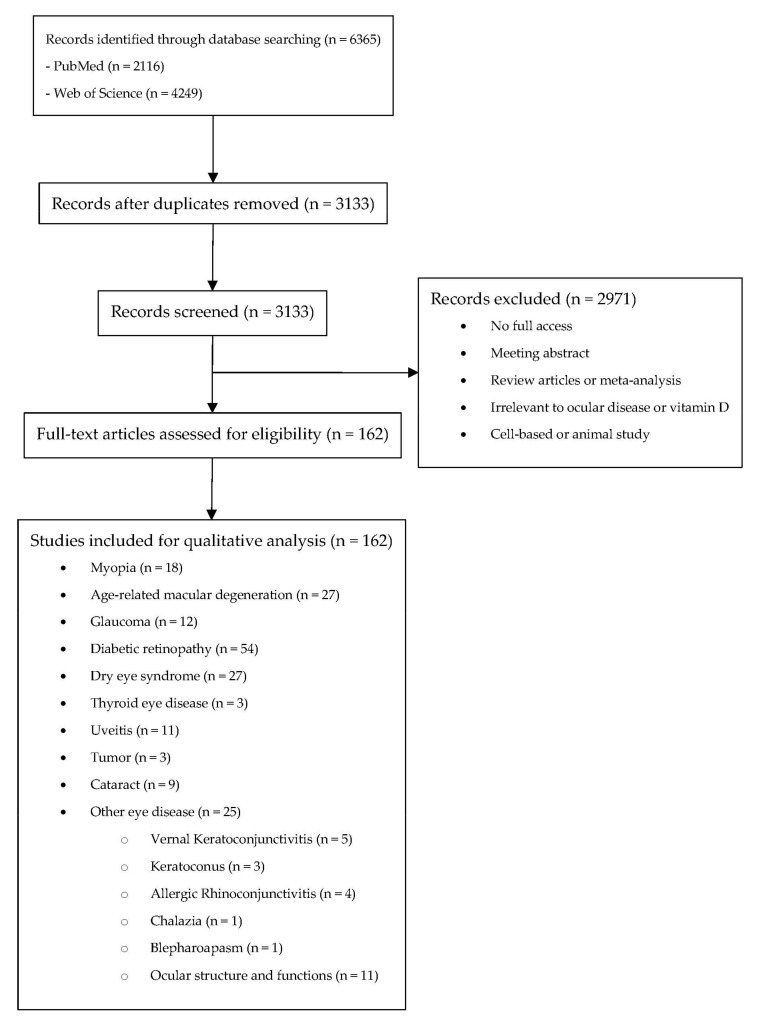
Systematic review flow diagram.

A systematic search on PubMed (Available online: http://www.ncbi.nlm.nih.gov/entrez/query.fcgi?DB5pubmed, accessed on 18 March 2022) and Web of Science (Available online: https://www.webofscience.com/wos/woscc/basic-search, accessed on 18 March 2022) with coverage up to 18 March 2022 was conducted initially using the following keywords: vitamin D in combinations with eye (PubMED: 544; WOS: 1909), eye disease (PubMed: 824; WOS: 854), ocular (PubMed: 183; WOS: 534), cataract (PubMed: 158; WOS: 355), lens opacity (PubMed: 118; WOS: 51), glaucoma (PubMed: 51; WOS: 151), intraocular pressure (PubMed: 24; WOS: 68), maculopathy (PubMed: 85; WOS: 97), diabetic retinopathy (PubMed: 126; WOS: 228), hypertensive retinopathy (PubMed: 2; WOS: 2), retinal arterial occlusion (PubMed: 0; WOS: 0), retinal venous occlusion (PubMed: 1; WOS:0). The search results from both databases were exported and imported in Covidence, which is a software for literature screening in systematic reviews. Among these 6813 results, the system detects that 3533 results were duplicated. They have been removed prior to the screening of the articles (Figure 2).

#### 3.1.2. Inclusion and Exclusion Criteria

The inclusion criteria for studies were: (1) written in English; (2) evaluating the association between blood vitamin D and different ocular diseases in a randomized controlled trial, prospective study, cross-sectional study, or case-control study. After a review of abstracts, relevant articles were retrieved and reviewed. Bibliographies of these articles provided further references. All retrieved records were reviewed by two independent reviewers (HNC and XL). Uncertainties were resolved via discussion with another reviewer (XJZ).

#### 3.1.3. Risk of Bias Assessment

Included interventional studies (both randomized controlled trials and clinical controlled trials) were assessed for quality according to the RoB tool for randomized trials from the Effective Practice and Organisation of Care (EPOC) Group. The assessment for the clinical controlled trial was assessed according to the suggestion from previous literature that both “random sequence generation” and “allocation concealment” were scored as “high risk”, while grading the remaining items as RCT [28]. We further modified the RoB tool by allocating 1 point to “low risk”, 0.5 point to “unclear risk” and 0 points to “high risk”. There are a total of 9 items to be assessed using the RoB tool, and hence, the total number of points for the RoB tool is 9 points, while those cohort, case-control, cross-sectional studies were assessed for quality according to the LEGEND (Let Evidence Guide Every New Decision) System designed for Cincinnati Children’s Hospital [29].

### 3.2. Myopia

Myopia is an important public health problem worldwide [30]. The etiology of myopia is complex, with both genetic and environmental risk factors [31,32,33,34]. Epidemiologic evidence indicates that time spent outdoors is a protective factor against myopia development [34,35,36,37,38], yet the underlying mechanism is unclear. Since the main source of vitamin D is sunlight exposure, vitamin D is linked to myopia, hypothesizing that a vitamin D pathway may mediate the protective effect of time spent outdoors on myopia. Evidence from studies on the relationship of vitamin D and myopia is summarized in Table 1.

As demonstrated in Table 1, the association between vitamin D and myopia is controversial in cross-sectional studies. Many studies suggest that the serum 25(OH)D3 level shows an inverse association with myopia and may have a protective effect on myopia [39,41,42,43,44,47,50,51,53,55]. However, several case-control studies from Australia [52], Denmark [46], and the US [54] found that the risks of myopia are not related to their neonatal vitamin D levels. 

Nevertheless, it is important to distinguish the causation between vitamin D and myopia. A large longitudinal cohort study found that 25(OH)D_3_ levels correlated with self-reported time spent outdoors; however, no evidence suggested that the participants’ serum vitamin D levels were independently associated with myopia [40]. Another study of preterm children also suggested that more time spent outdoors was associated with a lower risk of myopia, despite serum 25(OH)D_3_ concentrations not being shown to relate to myopia [48]. However, an Australian perspective study showed that, in young adults, myopia was most strongly associated with recent 25(OH)D3 concentrations, which is a marker of time spent outdoors [49].

Our meta-analysis found that the risk of myopia is inversely associated with blood 25(OH)D_3_ concentration after adjusting for sunlight exposure or time spent outdoors. However, this relationship was not significant among individuals under 18 years of age [57]. Polymorphisms in the vitamin D pathway genes may affect the development of myopia. One study reported the association of VDR polymorphisms, rs2853559, with myopia [58]. However, the results of other studies suggested that the true contribution of the vitamin D pathway to myopia could be negligible [42,45,59]. Our meta-analysis suggested that polymorphisms in the VDR gene are not associated with myopia [57]. On the other hand, animal studies proved that violet light (VL, *λ* = 360–400 nm) can suppress myopia progression, whereas no therapeutic effects were observed with UVB radiation (*λ* = 290–315 nm) [60], suggesting that UVB exposure and its dependent vitamin D synthetic pathway may not have a protective effect on myopia progression.

In conclusion, from the literature evidence, we know that, although blood 25(OH)D_3_ concentration is inversely associated with the risk of myopia, it seems unlikely that vitamin D has a direct protective effect on myopia progression. Instead, vitamin D levels may only serve as a biomarker for outdoor exposure.

### 3.3. Age-Related Macular Degeneration

As a chronic, progressive, degenerative disease, age-related macular degeneration (AMD) is a major cause of central blindness among people aged 60 years or over worldwide [61,62]. Oxidation, inflammation, and angiogenesis contribute to the pathogenesis of AMD, resulting in the dysfunction of RPE [63], Bruch’s membrane, and choriocapillaries [64]. In an aging retina, the complement cascade [65,66] and the tissue resident macrophage (retinal microglia) activation pathway [67] ultimately cause protein damage and aggregation, and degeneration of the RPE [68]. Angiogenesis, often caused by oxidative stress and inflammatory reactions, plays a major role in the development and progression of exudative AMD, potentially leading to severe and permanent visual impairment. 

The results of studies on cell lines and animal models have shown that vitamin D can protect cells or reduce oxidative stress [69,70,71]. Vitamin D has an anti-inflammatory role in chronic inflammatory diseases by decreasing the proliferation of T-cells and the production of pro-inflammatory agents [72,73]. On the other hand, vitamin D exerted an inhibitory effect on the angiogenesis signaling pathway [74,75], which may play a protective role in exudative AMD development and/or progression. Morrison et al. studied the variants in the vitamin D catabolizing enzyme, CYP24A1, and reported that variants (rs1570669, rs1570670, rs2274130, rs2296239, and rs4809957) were associated with reduced risk for AMD [76]. 

Table 2 summarized the studies on vitamin D and AMD. Case-control studies with small sample sizes suggest that AMD patients always have relatively low levels of serum vitamin D [77,78,79,80,81,82,83,84,85,86,87], except in a Iranian study, which did not find any significant correlation between serum vitamin D level and AMD [79]. However, this association seems to change in cross-sectional studies with larger sample sizes. Population-based studies held in France [88], the United States [89,90], and Israel [91] did not support a specific role for vitamin D in AMD, but vitamin D may work in some specific populations. An analysis of a sample of 1313 US participants indicated that high serum 25(OH)D3 concentrations may protect against early AMD in women less than 75 years old [92], while another US study supported the fact that levels of serum vitamin D were inversely associated with early AMD but not advanced AMD [93]. A Korean study had 17,045 participants and found that a high level of vitamin D was inversely associated with late AMD in men but not women [94]. Vitamin D deficiency in the European population was found to be associated with nvAMD, but the adjusted OR was small, and cannot exclude residual confounding [95].

Prospective studies, however, have not found a consistent association between vitamin D and the risk of developing AMD. In a large prospective cohort study of 2146 participants with a mean follow-up time of over 9 years, high dietary intake of vitamin D was significantly associated with a 40% lower risk of progression to advanced AMD [99]. However, recently, a nationwide, placebo-controlled, randomized clinical trial found that supplementing vitamin D had no significant overall effect on AMD incidence or progression in healthy people [98]. For this trial, 25,871 participants with a median age of 67.1 years were divided into four groups, receiving vitamin D supplements (2000 IU/day), ω-3 fatty acids (1 g/day), a combination of both, and placebo, respectively. After a median follow-up period of 5.3 years, no significant differences were found in the incidence or progression of AMD when compared with baseline [98]. This study suffered from a lack of stratification by clinical manifestations of AMD, a relatively short follow-up period for chronic disease, and a reliance on self-reported AMD diagnosis, leading to inconsistencies with the previous two studies [98,104].

In summary, cross-sectional studies suggest that vitamin D may have a protective effect on AMD formation, but this effect is small or may only work in a specific population. Furthermore, evidence from prospective cohort studies showed that continuously supplementing vitamin D may not reduce the risks of AMD over a period of several years.

### 3.4. Glaucoma

A leading cause of irreversible blindness, glaucoma is a group of optic neuropathies involving the death of retinal ganglion cells (RGCs) and the loss of their axons [105,106]. Two cross-sectional studies in South Korea reported that vitamin D deficiency is associated with glaucoma [107,108]. Similarly, a Chinese study found that the vitamin D deficiency, along with the presence of the BsmI ‘B’ allele and TaqI ‘t’ allele of the VDR gene, are relevant risk factors for glaucoma development [109]. Other studies in France [110], Croatia [111], the United States [112,113], and Turkey [114] have demonstrated that glaucoma patients have lower serum vitamin D levels compared to normal controls. However, another Turkish case-control study found no statistically significant difference in serum vitamin D levels between glaucoma patients and control subjects [115]. Similarly, a recent large-sample study in the United States showed that dietary intake, supplements, and serum levels of vitamin D are not significantly related to the risk of glaucoma [116]. Notably, ethnicity may contribute to the pathogenesis of glaucoma, giving rise to different conclusions among these studies [117]. Most of the literature reported the association between vitamin D and glaucoma and that a lower vitamin D concentration was found in glaucoma patients when compared with the control group [108,109,110,111,114], however, there were no findings on the association between vitamin D and the severity. Increases in vitamin D were associated with lower risks of having glaucoma (fourth quintile versus first quintile, OR 0.713, 95% confidence interval, 0.520 to 0.979) [108]. Only a limited study reported no statistically significant difference between the glaucoma group and the control group [108]; significantly lower vitamin D can only be found in advanced glaucoma patients [112] (Table 3).

High intraocular pressure (IOP) is an important risk factor for glaucoma. In an animal study on non-human primates, vitamin D treatment modulated the expression of IOP–regulating genes, with IOP falling in a dose-dependent manner [121]. However, a human study found no association between serum 25(OH)D_3_ levels and IOP, nor significant changes in participants’ IOP levels after receiving 6 months of oral vitamin D supplements (20,000 IU twice weekly) compared to the placebo group [118]. This contradiction may be due to the oral intake of vitamin D, which may lower the availability of vitamin D in the eye. Patients with glaucoma were found to have lower 25(OH)D concentrations in aqueous humor [119], and the IOP values were higher in cases of vitamin D deficiency [120]. Further studies are required to determine if vitamin D can be a potential intervention for glaucoma, especially through testing different supplement approaches. 

Some studies have identified vitamin D as an independent risk factor for glaucoma; however, the role that vitamin D plays in relation to glaucoma remains uncertain. Apart from the elevated IOP pathway, vitamin D may participate in the oxidative stress pathway due to its anti-oxidation and anti-inflammatory abilities. In an in vivo study, 1,25(OH)_2_D_3_ ameliorated the effects of oxidative stress from hydrogen peroxide-induced toxicity in human RPE cells through antioxidant signaling pathways, leading to lower levels of reactive oxygen species (ROS), cytokines, and vascular endothelial growth factor (VEGF) [122]. Another study demonstrated that vitamin D significantly altered the inflammatory-related genes in glaucoma, suppressing the expression of the angiotensin I–converting enzyme (ACE), carbonic anhydrase (CA), and Ras homologue gene family member A (RhoA), while significantly increasing the expression of the cytokine A20 precursor (CCL20) in the small intestines of rats [123]. ACE inhibitors are neuroprotective for cultured retinal neurons and can lower IOP in humans [124,125], while CA inhibitors can lower IOP and increase blood flow in the retinal vasculature and optic nerve [126]. The suppression of RhoA through subsequent vitamin D treatment can reduce aqueous outflow resistance and enhance fluid outflow [127,128]. Lastly, CCL2, an intraocular pressure responsive cytokine, possesses a potential role in intraocular pressure regulation [129].

In summary, all reported studies are cross-sectional studies (case-control studies and population surveys) and suggested the protective associations of vitamin D on glaucoma. Future studies should employ randomized clinical trial designs to investigate the causal relationship between glaucoma and low vitamin D levels or calcitriol deficiency.

### 3.5. Diabetic Retinopathy

Because of its ability to inhibit neovascularization, vitamin D has been studied in the development of diabetic retinopathy (DR). Many observational studies have examined the relationship between vitamin D levels and the prevalence or severity of DR, with most identifying an inverse association with both type 1 and 2 diabetes [130,131,132,133,134,135,136,137,138,139,140,141,142,143,144,145,146,147,148,149,150,151,152,153,154,155,156,157,158,159,160,161,162,163,164,165]. However, a Chinese study has reported a lack of association between vitamin D deficiency and DR after adjusting for all potential covariates, such as demographics, physical measurements, laboratory measurements, related complications, comorbidities, and medications [166]. Another Indian study suggested a possible association of vitamin D deficiency with type 2 diabetes, but not specifically with DR [167]. As demonstrated in Table 4, in general, some of the studies reported an inverse correlation between the serum vitamin D and severity of retinopathy [130,132,133,134,135,136,137,138,139,152,157,168,169]; similar findings were also reported, for example, the co-existence of low vitamin D and microvascular complications [131] or the association between the severity of DR and the prevalence of vitamin D deficiency [133,161,165] (Table 4); while some studies reported either no association or no significant difference between DR patients and healthy controls [170,171,172,173]. The agreement of the association between vitamin D deficiency and neuropathy is lower when compared with retinopathy. While some studies report that the risk for having diabetic neuropathy is higher in those with vitamin D deficiency [134,137], there is limited research on contrasting findings [174]. Further investigations are warranted.

Besides cross-sectional studies, a population-based prospective study also showed that a high level of vitamin D was associated with a lower risk of DR after 3 years [178]. A double-blind, placebo-controlled trial found that low blood 25(OH)D_3_ levels were associated with an increased risk of macrovascular and microvascular disease events among type 2 diabetics [139]. 

DR is a serious microvascular complication of diabetes. The characteristics of early DR include the loss of pericytes from retinal capillaries, the appearance of acellular capillaries and microaneurysms, and the breakdown of the blood-retinal barrier. In the proliferative phase of DR, neovascularization in the retina may occur, which significantly increases the probability of vision loss [184,185]. Potential mechanisms that explain how vitamin D can prevent DR include insulin resistance, immune regulation, anti-inflammation, and anti-angiogenesis. Animal studies have shown that vitamin D is important for insulin synthesis and can improve the body’s sensitivity to insulin, reducing the risk of insulin resistance [186,187]. Other studies have found that vitamin D treatment decreased the retinal expression of VEGF and the transforming growth factor TGF-β1 in rats [188], which may have protective effects on the retina. VDR has also been implicated in the pathogenesis of DR [189]. A meta-analysis of seven studies evaluating the association of the *VDR* gene polymorphisms with DR found that the FokI polymorphism of the *VDR* gene has a significant association with DR susceptibility [190]. Apart from the *VDR* polymorphism, other studies have proposed different protective mechanisms of vitamin D on DR, including protecting the vasculature [191,192,193], reducing oxidative stress [194,195], modulating inflammation and immune responses [180,196,197,198], inhibiting the renin-angiotensin aldosterone system [199,200], reducing the effects of advanced glycation end products [201,202], reducing endoplasmic reticulum stress [203,204], regulating endothelial cells apoptosis [205], and regulating diabetic leukostasis [206]. Further studies are needed to determine the exact mechanisms of vitamin D on DR.

In summary, even though there are no consistent associations between vitamin D level and DR in observational studies, more than 30 reports suggested an inverse relationship. The same conclusion is made in perspective studies, although the causal relationship has not been identified. Further studies should investigate whether vitamin D supplementation can reduce the risk of DR. 

### 3.6. Dry Eye Syndrome

Dry eye syndrome (DES), or dry eye disease [207], is a common eye disease affecting about 12% of the world’s population; prevalence was lowest in North America (4.6%) and highest in Africa (47.9%) [208]. Many factors are related to DES, including hormonal alterations, environmental changes, and aging [209]. DES is accompanied by the inflammation of the ocular surface, which may cause visual disturbances, tear film instability, and potential damage [210]. Whereas an increasing number of studies have shown that a relationship exists between vitamin D and DES, their findings remained controversial. Some cross-sectional studies suggested an inverse correlation between vitamin D levels and ocular surface disease index (OSDI) scores or DES incidence [211,212,213,214,215,216,217,218,219,220,221,222,223,224,225,226], while three others have not reached a significant conclusion [227,228,229,230], as demonstrated in Table 5. 

Several clinical trials investigated the treatment effects of vitamin D supplementation on DES symptoms, as demonstrated. Some of them only involved DES patients [232,236,237], while four other studies set healthy controls [219,231,233,235]. All these studies concluded that vitamin D can improve the DES symptoms of tear quality and ocular surface conditions. However, these studies may suffer from a small sample size or lack of a placebo-control group. Further well-design clinical trials with more samples are required to better understand the relationship between vitamin D and DES.

The key mechanism of vitamin D on DES may involve its antioxidation, anti-inflammatory, and immune-regulatory effects [239,240,241]. Vitamin D deficiency may cause the inflammation of the ocular surface and ultimately DES [233]. Conversely, vitamin D may relieve DES through inhibiting the interleukin-6 (IL-6) inhibitor [233], the key mediator of localized inflammation [242]. Moreover, vitamin D can suppress the release of inflammatory cytokines and stimulate the release of antioxidant cytokines in tears. Lastly, vitamin D can improve corneal epithelial barrier functions [233], which may improve ocular conditions. Apart from the role of vitamin D in the pathogenesis of DES, a study reported that the expression of VDR and CYP27B1 (vitamin D metabolism enzyme) was significantly decreased in DED patients, suggesting the possible involvement of the vitamin D regulatory enzyme in protecting the human eye from dry eye [243]. A study on SNPs of the VDR gene *Apa-1*, *Bsm-1*, *Fok-1* and *Taq-1*, reported the association of *Apa-1* and *Taq-1* with the risk of DES [244].

In conclusion, the exact mechanisms of vitamin D on DES are unclear, but evidence from cross-sectional studies seems to suggest that vitamin D has a protective effect against DES. Limit evidence from clinical trials also suggests that vitamin D supplementation could help to improve DES symptoms, but a further placebo RCT is needed to verify this treatment effect.

### 3.7. Thyroid Eye Diseases

Thyroid eye disease (TED), also known as Graves’ ophthalmopathy (GO), is an autoimmune inflammatory disorder. Few studies have examined the relationship between vitamin D and TED (Table 6). A pilot study in Texas, United States found prevalence rates of 20% and 31% for vitamin D deficiency and insufficiency among TED patients, respectively [245]. Another retrospective case-control study comparing vitamin D levels between Graves’ disease patients and TED patients found that low serum vitamin D was associated with TED [246]. Assessing and supplementing vitamin D levels may be an important addition to the early management strategies of GD patients. Since vitamin D can regulate immune responses and reduce inflammation, there is a definite need to further evaluate the role of vitamin D deficiency in TED patients. The current evidence for TED suggests that vitamin D would be associated with TED, however, the evidence is limited. It is worthwhile to further investigate the effect of vitamin D on TED.

### 3.8. Uveitis

Uveitis is the inflammation of the uvea driven by the T-cells [248]. It can be described as a failure of the ocular immune system, with the disease resulting from inflammation and tissue destruction. Since vitamin D can inhibit inflammation, influence T-cell responses, and regulate the immune system, it is necessary to examine its involvement in the development of uveitis. In an experimental autoimmune uveitis model, the oral administration of calcitriol was found to prevent and reverse the progression of uveitis by reducing the immunological response [249].

Although in a population-based study, none of the 25 uveitis patients were found to have vitamin D deficiency [250], two large retrospective case-control studies in the United States found an association of lower vitamin D levels with uveitis and scleritis, respectively [251,252]. Other case-control studies have been conducted on patients with specific types of uveitis, such as anterior uveitis (AAU) or non-infectious anterior uveitis [253,254,255], Vogt-Koyanagi-Harada (VKH) disease [256], sarcoidosis-associated uveitis [257], and juvenile idiopathic arthritis (JIA)-associated uveitis [258] (Table 7). All these studies suggested that the vitamin D deficiency is associated with uveitis development. A recent prospective case-control study consistently reported the association of vitamin D levels with active and inactive non-infectious uveitis patients. Vitamin D levels are related to uveitis severity [259].

There are reported associations between *VDR* polymorphisms and uveitis. Single-nucleotide polymorphisms (SNPs) of the *CYP2R1*, *CYP27B1*, *CYP24A1* and *DHCR7* genes are linked with lower circulating vitamin D levels [260,261,262,263]. A study in a Chinese cohort found that the frequencies of the genotype TT and T allele of *DHCR7* rs12785878 were both significantly higher among ocular Behçet disease (BD) patients compared with healthy controls; however, similar associations were not found for the VKH, AAU with ankylosing spondylitis (AS), or pediatric uveitis [263]. Another cross-sectional study found that gene variants involved in vitamin D anabolism and catabolism may be important for VKH pathology [25]. 

In summary, much evidence has shown that the onset and activity of uveitis are associated with vitamin D level, but most of the above studies were observational and did not provide evidence for causality. In the future, randomized-controlled studies are required to evaluate whether vitamin D can be an option for treating uveitis.

### 3.9. Retinoblastoma

Vitamin D has antineoplastic functions against many types of cancers through influencing cell differentiation, apoptosis regulation, anti-angiogenesis, and cell cycle arrest in various tumors [20]. Animal studies suggested that vitamin D analogues inhibited retinoblastoma (RB) tumor growth in athymic mice by increasing apoptosis, which is associated with the upregulation of both the p53 and p21 proteins [264,265]. However, due to the extremely low incidence rate of RB, few clinical studies have been conducted regarding the effect of vitamin D on RB. Two recent studies in Mexico found that sun exposure in early childhood protects against RB and may decrease the degree of intraocular spread in children with bilateral RB [266,267] (Table 8). However, RB is a childhood cancer, which usually presents at the age of 1 to 2 for bilateral and unilateral RB. Further studies are needed to confirm and understand the association between vitamin D levels and RB.

### 3.10. Cataract

For the past 20 years, the prevalence of cataracts has declined due to the advancement of surgical technology. However, in middle-income and low-income countries, cataract is still the most common cause of visual loss, accounting for 50% blindness [269]. Cataract is caused by losing lens transparency when the lens becomes opaque [269]. Epidemiology studies have shown that ultraviolet radiation is an important factor in increasing the risk of cataract [270,271,272]. Since the natural source of vitamin D is sunlight exposure, vitamin D may be involved in the pathophysiology of cataract. The opacity of the lens is a result of oxidative stress [273]. Since vitamin D can protect cells or reduce oxidative stress [69,70,71], it can be protective against cataract and play a role in lens metabolism.

There are studies attempting to reveal the association between vitamin D and cataract (Table 9). A large cross-sectional study in South Korea, with 16,086 participants aged 40 years or older, revealed that serum 25(OH)D levels were inversely associated with the risk of nuclear cataract [274]. Similarly, another South Korean study on 18,804 subjects also found that cataract risk decreased in men with higher serum 25-hydroxyvitamin D levels compared with those with lower serum 25(OH)D levels, but this association is not significant in women [275]. Other studies in Egypt [276], Turkey [277,278], and the UK [279] also showed that cataract patients often have a low level of serum vitamin D. However, one study [280] reported that serum 25(OH)D levels were not related to nuclear opacities. 

In summary, all the current studies are cross-sectional or retrospective in design, so the causal relationship between vitamin D and cataract needs to be proved by further large-prospective cohort or RCT. Cataract is essentially a treatable disease. Future study design should aim to find out whether vitamin D supplementation could protect elders against cataract development.

### 3.11. Other Ocular Diseases

Apart from the diseases described earlier, some studies reported on the association of vitamin D with curable or rare ocular diseases (Table 10). Vernal keratoconjunctivitis patients were found to be at a significantly lower level of serum vitamin D in case-control studies held in Turkey [284,285], Italy [286], and Iran [287]. Deficiency of vitamin D is also more frequently found in patients with keratoconus [288,289,290], retinal venous occlusions [291,292], and optic neuritis [293,294,295]. However, in children with allergic conjunctivitis, the results were contradictory: two studies [296,297] found significantly higher vitamin D levels in patients, while the other studies reported complete opposite conclusion [298,299]. 

It is notable that vitamin D is associated with most ocular diseases, from anterior segment to retina. One possible explanation is that vitamin D or its metabolite plays a role in maintaining the stability of ocular metabolism and structure. Higher 25(OH)D levels in aqueous humor may have an influence on ocular disease [119,283]. The change of ocular structure and function, including spatial contrast [300], contrast sensitivity [301], choroidal thickness [302,303,304], corneal endothelial [305], and macular thickness [306], may be affected by serum levels of vitamin D. On the other hand, this association is not a causality. Vitamin D may work as a marker of health status. People with poor health or low vision will have little outdoor activities and consequentially less exposure to sunlight. Some studies found a positive association or no association between vitamin D levels and pterygium [275,307,308,309,310]. Outdoor occupation is a major risk factor for the development of pterygium [311,312].

**Table 10:** Summary of studies related to other ocular diseases included.

First Author	Years	Country	Disease	Study-Design	Sample Size	Main Finding	Rate #
Gonul Karatas Durusoy [284]	2020	Turkey	VKC	Case-control study	46 VKC patients and 40 healthy controls	Children with VKC has a lower serum 25(OH)D_3_ levels when compared with healthy controls.	4b
Anna Maria Zicari [286]	2016	Italy	VKC	Prospective	47 VKC patients and 63 healthy controls	Children with VKC has a lower serum 25(OH)D_3_ levels when compared with healthy controls. And the vitamin D level was significantly correlated with the severity.	3b
Banu Bozkurt [285]	2016	Turkey	VKC	Case-control study	29 VKC patients and 62 healthy controls	Serum 25(OH)D_3_ levels in VKC children was significantly lower than those healthy control. 48.3% of VKC children and 22.6% healthy children were found to have severe vitamin D deficiency.	4b
Rana Sorkhabi [287]	2021	Iran	VKC	Case-control study	39 VKC patients and 32 healthy controls	Serum 25(OH)D_3_ levels in VKC patients were significantly lower than healthy controls. A statically insignificant reverse correlation of the serum vitamin D levels and the severity were found.	4a
Daniele Giovanni Ghiglioni [313]	2019	Italy	VKC	Prospective	71 VKC patients (mixed, 46; tarsal, 19; and limbal, 6)	There was a significant different in serum 25(OH)D_3_ levels in children with limbal VKC and tarsal VKC. The ocular treatment with immunomodulator eye drops allow the improvement in serum 25(OH)D_3_ levels.	3b
Mehmet Gökhan Aslan [288]	2021	Italy	KCN	Case-control study	28 progressive KCN patients, 27 nonprogressive KCN patients and 30 healthy controls	Serum 25(OH)D_3_ levels in KCN were significantly lower than healthy controls. Decreased vitamin D levels significantly increased nonprogressive KCN and progressive KCN probability 1.23 and 1.29 times, respectively.	4a
Serkan Akkaya [289]	2020	Turkey	KCN	Case-control study	100 KCN patients and 100 healthy controls	Serum 25(OH)D_3_ levels were significantly lower in KCN group than healthy control group, but no significant difference in the distribution of vitamin D levels among KCN groups of different severity.	4a
Siamak Zarei-Ghanavati [290]	2020	Iran	KCN	Cross-sectional	100 KCN patients and 100 healthy controls	A lower serum 25(OH)D_3_ level was found in the KCN group compared to the control group, but insignificant differences were found among different KCN stage groups.	4a
Sevil Bilir Goksugur [296]	2015	Turkey	ARC	Case-control study	22 ARC patients and 31 healthy controls	Serum 25(OH)D_3_ levels were associated with ARC in children.	4a
Alper Yenign [298]	2015	Turkey	ARC	Prospective cross-sectional study	42 ARC patients and 35 healthy controls	Plasma 25(OH)D_3_ levels in ARC patients were significantly lower than the control group.	4a
Zeynep Dadaci [299]	2014	Turkey	Seasonal ARC	Case-control study	49 seasonal ARC patients and 44 healthy controls	Plasma 25(OH)D_3_ levels of seasonal ARC were significantly lower than control group.	4a
Farhan Khashim Alswailmi [297]	2021	Saudi Arabi	Seasonal ARC	Cross-sectional case-control study	26 seasonal ARC patients and 26 healthy controls	Mean vitamin D level was significantly higher in seasonal ARC patients. Higher serum vitamin D levels may be linked with seasonal ARC.	4b
Lin Chen [314]	2014	China	Chalazia	Prospective case-control study	88 chalazia patients and 72 healthy controls	No significant differences in vitamin D_3_	4b
Kubra Serefoglu Cabuk [26]	2020	Turkey	Blepharospasm	Prospective case-control study	50 Blepharospasm patients and 22 healthy controls	Serum 25(OH)D_3_ levels were moderately negatively correlated with Blepharospasm severity (Jankovic severity score).	4b
Emrah Utku Kabatas [315]	2017	Turkey	Retinopathy	Prospective	97 premature infants patients	Serum 25(OH)D_3_ levels were significantly lower in infants with retinopathy of prematurity than those without retinopathy of prematurity.	3a
Sedat Arikan [300]	2020	Turkey	Spatial contrast	Prospective	41 VDD patients and 30 without VDD controls	Vitamin D deficiency cause a decrease in contrast sensitivity function.	3a
Emrah Ozturk [301]	2020	Turkey	Contrast sensitivity	Prospective	42 VDD patients and 34 normal levels control	VDD had negative effects on contrast sensitivity function and also caused thickness difference in certain segments of retinal layers.	3a
Aydemir Gozde Aksoy [302]	2022	Turkey	Choroidal thickness	Case-control study	46 DM with VDD patients, 42 DM with normal vitamin D level patients, and 73 healthy controls	No difference in retinal nerve fibre layer (RNFL) between three groups. VDD has no effect on the RNFL. However, a positive correlation existed between the macular choroidal thickness (CT) and the vitamin D levels in DM patients with VDD.	4a
Esra Vural [303]	2020	Turkey	Choroidal thickness	Prospective case-control study	30 VDD patients and 30 normal level controls	A positive correlation was found between vitamin D levels and subfoveal choroidal thickness and inferior and nasal peripapillary choroidal thickness in all participants.	4a
Hasan Oncul [304]	2020	Turkey	Choroidal thickness	Prospective case-control study	65 VDD patients and 60 normal level controls	VDD patients have a thinner choroid and the choroidal thickness increased after vitamin D replacement therapy.	4a
Cem Cankaya [305]	2018	United States	Corneal endothelial	Case-control study	58 VDD patients and 40 normal level controls	The mean corneal endothelial cell density and mean hexagonal cell ratio in VDD patients were lower than healthy controls. The mean coefficient of variation in VDD patients were higher than healthy controls. VDD may affect the corneal endothelial layer.	4a
Alix Graffe [306]	2014	France	Macular thickness	Cross-sectional	62 patients (17 and 45 with vitamin D insufficiency and sufficiency, respectively)	Vitamin D insufficiency was associated with reduced macular thickness with no patent macular dysfunction.	4a
Unal Mutlu [316]	2016	Netherlands	Retinal microvascular	Prospective population-based study	5675 subjects (sample) and 2973 subjects (subsample)	Individuals with lower vitamin D levels were more likely to have retinopathy. Lower vitamin D levels were associated with wider venular calibers.	3a
Hatice Daldal [317]	2021	Turkey	Ocular findings	Prospective	98 patients (41, 45 and 12 were vitamin D severe deficient, deficient and insufficient, respectively)	Vitamin D may be related to thinning in macular and nasal of RNFL.	3a
Karabulut Mujdat [318]	2022	Turkey	Retinal microvascularity	Case-control study	98 VDD patients and 96 healthy controls	There was a strong negative correlation between the serum vitamin D level and vessel density in the whole image, parafoveal, and perifoveal regions of the deep capillary plexus in the study group (Spearman’s rho = −0.71, *p* = 0.043; Spearman’s rho = −0.79, *p* = 0.011; and Spearman’s rho = −0.74, *p* = 0.032; respectively).	4a

VKC: vernal keratoconjunctivitis; KCN: keratoconus; ARC: allergic rhinoconjunctivitis; VDD: vitamin D deficiency; # LEGEND for case-control, cohort, and cross-sectional studies, rating of the studies follow the guidelines from LEGEND.

## 4. Perspective

In this systematic review, we summarize the evidence of vitamin D’s effect on different ocular diseases. However, there are significant limitations in many studies, and the interpretation of results should be within these limitations. 

First, the definition of vitamin D deficiency is not consistent in different studies, which affects the objective comparison of results from different studies. Until 1998, vitamin D deficiency was defined as a blood level of 25(OH)D, which represents a total concentration of both 25-hydroxyvitamin D2 and 25-hydroxyvitamin D3 of less than 10 ng/mL (25 nmol/L). However, this definition was redefined in 1998 as a blood level of 25(OH)D < 20 ng/mL (50 nmol/L). One reason for the change in the involvement of PTH: adults may require a serum 25(OH)D of at least 50 nmol/L to achieve optimum PTH levels [319]. Moreover, some studies use quintiles or quartiles to perform data analysis. We suggest that future studies should use the common classification to analyze serum vitamin D levels. 

Second, vitamin D levels are related to different socioeconomic, lifestyle, and dietary factors. These factors are very important when considering the onset or development of ocular diseases. Future studies should determine these factors and consider them as confounders in statistical analysis. 

Finally, few studies provided causal evidence on whether supplementing vitamin D can reduce the prevalence of ocular diseases. Currently, available evidence is insufficient to confirm how vitamin D works in ocular diseases. Moreover, investigations of those metabolic enzymes are also important to understand why significantly low vitamin D is found in patients with ocular diseases. In summary, among all ocular diseases, the association between vitamin D and AMD was unclear, while clinical trials in DES or DR required bigger sample sizes in different ethnic populations. Other ocular diseases such as cataract, myopia, and uveitis, no RCTs or perspective cohorts have been reported, so their associations with vitamin D are still unclear. 

## 5. Vitamin D and Eye Care

Vitamin D can be a potential intervention for different ocular diseases. Although findings for certain ocular diseases are inconsistent, they can still serve as references for further studies that examine the therapeutic effects of vitamin D on ocular diseases, considering that vitamin D deficiency is a common health issue worldwide, and vitamin D has wide safety doses and rare side effects. Even though more evidence from the randomized controlled trial is needed to confirm the effect of vitamin D on various ocular diseases, it is recommended to maintain blood 25(OH)D_3_ at a desirable level (25–50 nmol/L) by spending a short period of time outdoors, baring skin to the sun, and boosting vitamin D intake by a daily supplement of 400–800 international units (10 to 20 μg). 

The human body can also obtain vitamin D naturally from sunlight exposure, however, both extended exposure to unprotected sunshine, which also increases the risk for cataracts, and AMD and completely avoiding sunlight by applying UV B sunscreen should be avoided. Several factors affect vitamin D production, and a more efficient production of vitamin D can be achieved when someone is closer to the equator, has a lighter skin color, and/or exposes larger surfaces of skin during summer midday without sunscreen. Generally, 5–30 min of sun exposure on the unprotected face, arms, legs, or back between 10 a.m. and 3 *p*.m. twice to three times a week is enough for sufficient vitamin D. Even though vitamin D can be beneficial to our ocular health, long term prolonged sun exposure is also associated with corneal sunburn, tissue growths on sclera, cataracts, and macular degeneration, and wearing hats, and sunglasses are recommended to protect the eyes from UV damage.

## 6. Conclusions

Various epidemiological and clinical studies have demonstrated a connection between vitamin D deficiency and ocular diseases, such as myopia, age-related macular degeneration, glaucoma, diabetic retinopathy, dry eye syndrome, thyroid eye disease, uveitis, retinoblastoma, and cataract, among others. While vitamin D is associated with potential pathways related to these respective diseases, their pathogeneses are complicated, and current understandings of the underlying mechanisms remain limited. Vitamin D not only affects mineral metabolism homeostasis, but also possesses antioxidation and anti-inflammatory properties. It also plays a role in anti-angiogenesis, modulating cell cycle including cell proliferation, differentiation, and apoptosis. VDR and vitamin D regulatory enzymes are present in ocular tissues, and studies have demonstrated that ocular tissues can activate and regulate vitamin D, suggesting the importance of vitamin D in maintaining ocular health. 

## Figures and Tables

**Table 1:** Summary of studies related to myopia included.

First Author	Years	Country	Study Design	Sample Size	Main Finding	Rate #
Jin A. Choi [39]	2014	South Korea	Cross-sectional study	2038 subjects	Individuals with higher concentrations of serum 25(OH)D had lower prevalences of myopia after adjustment for confounding factors (*p* < 0.001). In multiple linear regression analyses, spherical equivalent was significantly associated with serum 25(OH)D concentration after adjustment for confounding factors (*p* = 0.002).	4a
Jeremy A. Guggenheim [40]	2014	UK	Prospective observational study	3677 subjects	Total vitamin D and D3 were biomarkers for time spent outdoors, however there was no evidence they were independently associated with future myopia.	3a
Donald O. Mutti [41]	2011	United States	Cross-sectional study	32 subjects	Adjusted for differences in the intake of dietary variables, myopes appear to have lower average blood levels of vitamin D than non-myopes.	4b
J. Willem L. Tideman [42]	2016	Netherlands	Cross-sectional study	2666 subjects	Serum levels of 25(OH)D_3_ were inversely related to AL, and that low levels increased the risk of myopia. This relationship may be independent from time spent outdoors.	4a
Seyhan Yazar [43]	2014	Australia	Cross-sectional study	946 subjects	Myopic participants had significantly lower 25(OH)D_3_ concentrations. The prevalence of myopia was significantly higher in individuals with vitamin D deficiency compared to the individuals with sufficient levels.	4a
Jin-woo Kwon [44]	2017	South Korea	Cross-sectional study	15,126 subjects	Low serum 25(OH)D_3_ levels and shorter daily sun exposure time may be independently associated with a high prevalence of myopia in Korean adults. These data suggest a direct role for vitamin D in the development of myopia.	4a
Katie M. Williams [45]	2016	Europe including Norway, Estonia, United Kingdom, France, Italy, Greece, and Spain	Cross-sectional study	3168 subjects	No independent associations between myopia and serum vitamin D3 concentrations nor variants in genes associated with vitamin D metabolism were found. No convincing evidence for a direct role of vitamin D in myopia risk.	4a
Ina Olmer Specht [46]	2020	Denmark	Case-control study	457 myopic subjects and 1280 emmetropic subjects	No increased odds of myopia in relation to low neonatal 25(OH)D3 levels, and no seasonal variation in myopia risk.	4a
Byung J Jung [47]	2020	South Korea	Cross-sectional study	25,199 subjects	Serum 25(OH)D_3_ level was inversely associated with myopia in Korean adults.	4a
Hung-Da Chou [48]	2021	China	Prospective, cross-sectional study	99 Preterm children	Among preterm children with or without ROP, more time spent outdoors was associated with lower odds of myopia. The serum 25(OH)D_3_ concentration was not associated with myopia, but a high proportion of the participants had insufficient levels.	4a
Gareth Lingham [49]	2021	Australia	Multi-generation, longitudinal cohort study	1260 subjects	Myopia in young adulthood was most strongly associated with recent 25(OH)D_3_ concentrations, a marker of time spent outdoors.	3a
Sang Beom Han [50]	2019	South Korea	Cross-sectional study	3398 subjects	Lower serum 25(OH)D_3_ concentration (<9 ng/mL) was associated with increased prevalence of both myopia and high myopia. Serum 25(OH)D_3_ concentration of ≥9 ng/ml was significantly associated with decreased prevalence of high myopia in participants with near work of ≥3 h/day, although the effect was not significant in myopia and low myopia.	4a
Fan Gao [51]	2021	China	Cross-sectional study	186 subjects	Children with a higher level of serum 25(OH)D_3_ have a lower prevalence of moderate to high myopia.	4a
Gareth Lingham [52]	2019	Australia	Cross-sectional study	4112 subjects	Vitamin D levels are unrelated to myopia risk in adults.	4a
Ho Sik Hwang [53]	2018	South Korea	Cross-sectional study	11,703 subjects	Serum 25(OH)D_3_ level (OR, 0.97 per 1 ng/mL) showed protective effect against high myopia	4a
Harb Elise N [54]	2021	United States	Cross-sectional study	4838 subjects	There is nonsignificant correlation between serum vitamin D levels and spherical equivalent refractive errors	4a
Didik Wahyudi [55]	2020	Indonesia	Non-randomised pre-post study	80 subjects	Vitamin D supplementation and sunlight exposure could increase serum 25-hydroxyvitamin D, decrease myopia, and inhibit the progression of myopia.	8/12*
Xiaoman Li [56]	2022	China	Cross-sectional study	294 lowland area children and 89 highland area children	There is no association between serum 25(OH)D concentration and myopia in the 6–14 years old Chinese children.	4a

* NIH quality assessment tool for before-after (Pre-Post) study with no control group; # LEGEND for case-control, cohort, and cross-sectional studies, rating of the studies follow the guidelines from LEGEND.

**Table 2:** Summary of studies related to age-related macular degeneration included.

First Author	Years	Country	Study-Design	Sample Size	Main Finding	Rate #
Audrey Cougnard-Grégoire [88]	2018	South Korea	Case-control study	32 Early AMD, 30 late AMD, and 34 normal controls	Serum vitamin D deficiency increases the risk of early AMD with borderline significance [odds ratio (OR) = 3.59; 95% confidence interval (95% CI) 0.95–13.58; *p* = 0.060], while significantly associated with a higher risk of late AMD (OR = 3.61; 95%CI 1.04–12.51; *p* = 0.043). In 2 subgroups of late AMD, serum vitamin D deficiency only increase the risk of patients with subretinal fibrosis (OR = 7.54; 95% CI 1.34–42.51), but not. However, there was no significant association between serum vitamin D deficiency and late AMD without subretinal fibrosis (OR = 1.89; 95% CI 0.40–8.92).	4a
Shelley Day [89]	2017	Europe (Norway, Estonia, UK, France, Italy, Greece, Spain)	Cross-sectional study	2209 Early AMD, 150 late AMD and 104 nvAMD	No linear association was found with 25(OH)D and early or late AMD or nvAMD. Deficient status was associated with nvAMD (adjusted OR, 1.27; 95% confidence interval, 1.1–1.45; *p* < 0.0001), but no association between insufficient or deficient status with early or late AMD.	4b
S Golan [91]	2015	United States	Cross-sectional study	913 subjects	For women with vitamin D deficient (<12 ng/mL), there were 6.7-fold increased odds of AMD (95% CI, 1.6–28.2).	4b
Alix Graffe [78]	2019	United States	Prospective study	1225 subjects	High 25(OH)D_3_ concentrations, approximately >70 nM, may be associated with decreased odds of incident early AMD.	4b
Rezvan Hashemi [79]	2017	United States	Cross-sectional study	9734 subjects	The adjusted OR (95% CIs) for early AMD among those with adequate (=75 nmol/L) compared to deficient (<30 nmol/L) vitamin D status was 0.94 (0.59–1.50), *p*-trend = 0.86. Vitamin D status was not associated with early AMD in this cohort sample.	4b
Sujit Itty [96]	2011	United States	Cross-sectional study	1313 subjects	Serum 25(OH)D was associated with decreased odds of early AMD in women younger than 75 years and increased odds in women aged 75 years or older (OR for quintile 5 vs. 1, 0.52; 95% CI, 0.29–0.91; *p* for trend = 0.02 and OR, 1.76; 95% CI, 0.77–4.13; *p* for trend = 0.05, respectively). High serum 25(OH)D_3_ concentrations may protect against early AMD in women younger than 75 years.	4a
Emrah Kan [80]	2011	United States	Cross-sectional study	100 subjects (50 pairs of siblings)	Comparing among affected and unaffected siblings, serum 25(OH)D levels were not statistically different (*p* = 0.22). Although evaluation of serum 25(OH)D_3_ was higher in unaffected individuals than in their affected siblings, but the finding did not reach statistical significance.	4a
Eun Chul Kim [94]	2007	United States	Cross-sectional study	7752 subjects	Levels of serum vitamin D were inversely associated with early AMD but not advanced AMD. The odds ratio (OR) and 95% confidence interval (CI) for early AMD among participants in the highest vs. lowest quintile of serum vitamin D was 0.64 (95% CI, 0.5–0.8; *p* trend <0.001).	4a
Kyoung Lae Kim [81]	2013	Denmark	Cross-sectional study	178 subjects	Across different AMD stages by CARMS, the plasema 25(OH)D levels were comparable. In CARMS 5, the presence of subretinal fibrosis was associated with significantly lower concentrations of 25-hydroxyvitamin D as compared to the absence of subretinal fibrosis (47.2 versus 75.6 nmol/L, *p* < 0.001). Patients in CARMS 5 with subretinal fibrosis were more likely to have insufficient levels of 25-hydroxyvitamin D compared to patients without subretinal fibrosis (*p* = 0.006)	4a
Gareth J.McKay [95]	2020	United States	RCT	25,871 subjects	Neither vitamin D3 nor marine ω-3 fatty acid supplementation had a significant overall effect on AMD incidence or progression.	4a
Amy E. Millen [92]	2017	France	Prospective cohort study	2146 subjects	Compared with the highest and lowest quintile of dietary vitamin D intake after adjustment for other confounding facters, there was a lower risk of progression to late AMD and NV (for late AMD: hazard ratio [HR]: 0.60; 95% confidence interval [CI]: 0.43–0.83; *p* trend = 0.0007; for NV: HR: 0.59; 95% CI: 0.39–0.89; *p* trend = 0.005) but not GA (HR: 0.83; 95% CI: 0.53–1.30; *p* trend = 0.35). When supplement use was considered, the effect was in the protective direction but was not significant. A diet rich in vitamin D may prevent or delay progression to advanced AMD, especially nvAMD.	4a
Amy E. Millen [87]	2016	Japan	Case-control study	161 Neovascular AMD patients and 369 healthy controls	Logistic regression analysis demonstrated that low intakes of vitamin D was associated with neovascular AMD (Trend *p* = 0.002 for vitamin D). High dietary intake of vitamin D is associated with a reduced risk of AMD.	3a
Amy E. Millen [90]	2011	France	Cross-sectional study	311 subjects	Low serum 25OHD concentrations were associated with poorer vision acuity.	4a
Amy E. Millen [97,98]	2021	Turkey	Retrospective study	114 ARMD and 102 healthy controls	The age-related macular degeneration group had significantly lower vitamin D levels than the control group (*p* > 0.001). Significantly decreased levels of 25(OH) vitamin D in advanced-stage age-related macular degeneration suggest a significant correlation existing between vitamin D deficiency and age-related macular degeneration development	4a
Margaux A. Morrison [76]	2020	Egypt	Cross-sectional study	222 Primary osteoarthritis patients (46 with AMD, 176 without AMD)	Less vitamin D intake were significantly associated with AMD occurrence in primary osteoarthritis patients.	4b
Niyati Parekh [93]	2019	Italy	RCT	30 Intermediate AMD	In intermediate AMD, Macuprev^®^ supplementation (contained vitamin D3 800 IU) increases the function of the macular pre-ganglionic elements, with no associated retinal and choroidal ultra-structural changes.	4a
Amardeep Singh [82]	2011	United States	Case series	184 Caucasian male twin pairs	Higher dietary intake of vitamin D was present in the twins with less severe AMD (*p* = 0.01) and smaller drusen size (*p* = 0.05) compared with co-twins, adjusted for smoking and age	4b
William G. Christen [99]	2015	France	Cross-sectional study	91 subjects	Patients with vitamin D deficiency (*n* = 11) had a reduced mean GCC thickness compared to those without vitamin D deficiency (72.1 ± 7.4 μm versus 77.5 ± 7.5 μm, *p* = 0.028)	9/9 * (RoB)
Bénédicte M. J. Merle [100]	2022	Spain	Cross-sectional study	93 AMD patients and 93 healthy controls	The AMD patients had statistically significant lower 25 (OH)D levels than healthy controls but the median 25(OH)D levels in different stages and subtypes (early, intermediate, advance atrophic and advanced neovascular) were not statistically significant.	3a
Aya Aoki [101]	2018	South Korea	Case-control study	32 Early AMD, 30 late AMD, and 34 normal controls	Serum vitamin D deficiency increase the risk of early AMD with borderline significance [odds ratio (OR) = 3.59; 95% confidence interval (95% CI) 0.95–13.58; *p* = 0.060], while significantly associated with a higher risk of late AMD (OR = 3.61; 95%CI 1.04–12.51; *p* = 0.043). In 2 subgroups of late AMD, serum vitamin D deficiency only increase the risk of patients with subretinal fibrosis (OR = 7.54; 95% CI 1.34–42.51), but not. However, there was no significant association between serum vitamin D deficiency and late AMD without subretinal fibrosis (OR = 1.89; 95% CI 0.40–8.92).	4a
Olivier Beauchet [102]	2017	Europe (Norway, Estonia, UK, France, Italy, Greece, Spain)	Cross-sectional study	2209 Early AMD, 150 late AMD and 104 nvAMD	No linear association was found with 25(OH)D and early or late AMD or nvAMD. Deficient status was associated with nvAMD (adjusted OR, 1.27; 95% confidence interval, 1.1–1.45; *p* < 0.0001), but no association between insufficient or deficient status with early or late AMD.	3a
Naciye Kabataş [83]	2015	United States	Cross-sectional study	913 subjects	For women with vitamin D deficient (<12 ng/mL), there was 6.7-fold increased odds of AMD (95% CI, 1.6–28.2).	4a
Marwa Yahia Mahgoub [86]	2019	United States	Prospective study	1225 subjects	High 25(OH)D_3_ concentrations, approximately >70 nM, may be associated with decreased odds of incident early AMD.	4a
Mariacristina Parravano [103]	2017	United States	Cross-sectional study	9734 subjects	The adjusted OR (95% CIs) for early AMD among those with adequate (=75 nmol/L) compared to deficient (<30 nmol/L) vitamin D status was 0.94 (0.59–1.50), *p*-trend = 0.86. Vitamin D status was not associated with early AMD in this cohort sample.	8/9 * (RoB)
Johanna M Seddon [84]	2011	United States	Cross-sectional study	1313 subjects	Serum 25(OH)D was associated with decreased odds of early AMD in women younger than 75 years and increased odds in women aged 75 years or older (OR for quintile 5 vs. 1, 0.52; 95% CI, 0.29–0.91; *p* for trend = 0.02 and OR, 1.76; 95% CI, 0.77–4.13; *p* for trend = 0.05, respectively). High serum 25(OH)D_3_ concentrations may protect against early AMD in women younger than 75 years.	4a
Mathieu Uro [85]	2011	United States	Cross-sectional study	100 subjects (50 pairs of siblings)	Comparing among affected and unaffected siblings, serum 25(OH)D levels were not statistically different (*p* = 0.22). Although evaluation of serum 25(OH)D_3_ was higher in unaffected individuals than in their affected siblings, but the finding did not reach statistical significance.	3a
Perez Serena [104]	2007	United States	Cross-sectional study	7752 subjects	Levels of serum vitamin D were inversely associated with early AMD but not advanced AMD. The odds ratio (OR) and 95% confidence interval (CI) for early AMD among participants in the highest vs. lowest quintile of serum vitamin D was 0.64 (95% CI, 0.5–0.8; *p* trend <0.001).	3a

nvAMD = nonvascular AMD; * The Effective Practice and Organisation of Care (EPOC) RoB Tool for randomized trials; # LEGEND for case-control, cohort, and cross-sectional studies, rating of the studies follow the guidelines from LEGEND.

**Table 3:** Summary of studies related to glaucoma included.

First Author	Years	Country	Study-Design	Sample Size	Main Finding	Rate #
Einar Andreas Krefting [118]	2013	Norway	1.Nested case-control; 2. randomized controlled intervention trial	1. 87 low serum vitamin D3 group and 42 healthy controls (high serum vitamin D3 group 2. 39 vitamin D and 39 placebo	Intraocular pressure in the 87 participants with low serum 25(OH)D levels (mean 40.1 ± 12.9 nm) did not differ from IOP in the 42 participants with high serum 25(OH)D levels (mean 85.1 ± 14.0 nm) (15.9 ± 3.3 mmHg versus 15.6 ± 3.1 mmHg, *p* = 0.56, independent t-test). No associations between serum 25(OH)D_3_ levels and IOP, and administration of vitamin D3 to participants with low levels of 25(OH)D_3_ did not affect IOP.	9/9 * (RoB)
Tae Keun Yoo [107]	2014	South Korea	Cross-sectional	290 POAG, 410 Glaucoma suspects and 5394 healthy controls	A reverse J-shaped association between 25(OH)D_3_ levels and the risk of OAG, with significantly elevated risk at lower 25(OH)D_3_.	4a
Aurélien Goncalves [110]	2015	France	Case-control	99 Severe POAG, 51 moderate POAG, and 164 healthy controls	Decreased serum 25OHD concentration was associated with POAG. There was no 25OHD difference between moderate and severe POAG.	4a
Hyun Tae Kim [108]	2016	South Korea	Retrospective cross-sectional study	1627 Glaucoma and 12,1704 healthy controls	In the multivariable-adjusted logistic analysis, the odds ratio of glaucoma in the fourth quintile was significantly lower than that of the first quintile in females (odds ratio, 0.713; 95% confidence interval, 0.520 to 0.979). Lower 25(OH)D level was significantly associated with an elevated risk of glaucoma in females compared with higher 25(OH)D level.	4a
Yingjuan Lv [109]	2016	China	Hospital-based and case-control study	71 POAG and healthy controls	The serum levels of 1 a, 25-Dihydroxyvitamin D3 in age-matched controls was significantly higher than the levels in POAG patients. (*p* < 0.001). Vitamin D deficiency and the presence of the BsmI ‘B’ allele and the TaqI ‘t’ allele are relevant risk factors in the development of glaucoma.	2a
Željka Vuković Arar [111]	2016	Croatia	Case-control	20 POAG and 20 healthy controls	Serum vitamin D level was statistically significantly lower in glaucoma patients as compared with control group. No statistically significant difference in the IOP values between glaucoma patients and control subjects.	2a
Radha Ayyagari [112]	2019	United States	Case-control	357 POAG and 178 healthy controls	Patients with advanced glaucoma had lower serum levels of vitamin D compared with early glaucoma and normal subjects.The mean (95% confidence interval [C]) levels of vitamin D of the subjects in the control (8.02 ± 6.19 pg/mL) and early phenotype (7.56 ± 5.74 pg/mL) groups were significantly or marginally significantly different from the levels observed in subjects with the advanced phenotype (6.35 ± 4.76 pg/mL; *p* = 0.0117 and 0.0543, respectively).	2a
Seyhan Dikci [115]	2019	Turkey	Prospective, cross-sectional study	31 PEX glaucoma, 34 PEX syndrome and 43 healthy controls	No statistically significant difference between the PEX syndrome/glaucoma, and control group in terms of serum vitamin D levels, serum vitamin D levels were lower in PEX syndrome and glaucoma group than control group.	3b
K.Atalay [114]	2019	Turkey	Prospective, cross-sectional study	29 XFG, 77 POAG and 33 healthy control	Mean vitamin D levels show a significant difference between the control group and POAG (*p* = 0.015)	3a
Yongwun Cho [119]	2021	South Korea	Case-control	25 OAG and 90 health controls	Patients with OAG had significantly lower 25(OH)D concentrations in aqueous humor than control patients	4a
Timur Ekiz [113]	2016	United States	Case-control	23 PEX glaucoma and 46 healthy controls	Although patients with ocular pseudoexfoliation have low bone mineral density and 25(OH)D, bone mineral density and 25(OH)D do not appear to be linked to ocular pseudoexfoliation syndrome in our study population.	2b
Tolga Kocaturk [120]	2019	Turkey	Cross-sectional	120 subjects	The IOPg values were higher in cases of vitamin D deficiency. Corneal biomechanical differences in patients with lack of vitamin D were not statistically significant.	3b

* The Effective Practice and Organisation of Care (EPOC) RoB Tool for randomized trials; # LEGEND for case-control, cohort, and cross-sectional studies, rating of the studies follow the guidelines from LEGEND.

**Table 4:** Summary of studies related to diabetic retinopathy included.

First Author	Years	Country	Study-Design	Sample Size	Main Finding	Rate #
HülyaAksoy [130]	2000	Turkey	Cross-sectional study	20 DM without DR patients, 15 DM with BDR patients, 14 pre-DM with PDR patients, 17 DM with PDR patients and 20 healthy controls	There was an inverse relationship between the severity of the retinopathy, neovascularization, and serum 1,25(OH)_2_D_3_ concentrations, being the lowest in PDR and the highest in diabetic patients without retinopathy patients. Mean 1,25(OH)2D3 concentrations fell with increasing severity of diabetic retinopathy. Only mean 1,25(OH)2D3 concentrations were not significantly different between NDR and BDR, pre-PDR and PDR (*p* > 0.05). Mean 1,25(OH)2D3 concentrations were significantly different among the other groups (*p* < 0.05).	3a
Atsushi Suzuki [131]	2006	Japan	Descriptive study	581 T2DM patients and 51 healthy controls	Serum concentration of 25(OH)D_3_ were associated with HbAlc (*p* = 0.013). Microvascular complications and insulin treatment in T2DM patients are associated with the co-existence of hypovitaminosis D although serum creatinine levels were below 2.0 mg/dl.	4a
Harleen Kaur [132]	2011	Australia	Cross-sectional study	517 T1DM patients	Vitamin D deficiency is associated with an increased prevalence of retinopathy in young people with T1DM. In logistic regression, retinopathy was associated with VDD (odds ratio 2.12 [95% CI 1.03–4.33]), diabetes duration (1.13, 1.05–1.23), and HbA1c (1.24, 1.02–1.50).	4a
Christel Joergensen [175]	2011	Denmark	Prospective observational study	227 T1DM patients	In patients with type 1 diabetes, severe vitamin D deficiency independently predicts all-cause mortality (unadjusted HR 2.0 [0.9–4.4], *p* = 0.1 and HR 2.7 [1.1–6.7], *p* = 0.03, respectively) but not development of microvascular complications in the eye (HR 1.1 [0.7–1.7], *p* = 0.8) and kidney (unadjusted HR 1.1 [0.5–2.4], *p* = 0.8 and HR 1.3 [0.3–5.4], *p* = 0.7, respectively).	3a
Patricia A. Patrick [133]	2012	United States	Cross-sectional study	1790 subjects	There is an association between severity of diabetic retinopathy and prevalence of vitamin D deficiency (β = 1.3, *p* = 0.01, unadjusted; and β = 1.2, *p* = 0.01, adjusted for age and obesity status given their clinical significance). However, regression analysis of these data did not demonstrate a statistically significant relationship between the 2 variables (β = −0.04, *p* = 0.07, unadjusted; and β = −0.03, *p* = 0.13, adjusted). The findings were inconclusive about the existence of a relationship between retinopathy severity and serum 25(OH)D_3_ concentration.	3a
John F Payne [176]	2012	Georgia	Cross-sectional study	221 subjects (17 No diabetes or ocular diseases; 51 No diabetes with ocular disease; 41 No BDR; 40 NPDR; 42 PDR)	Patients with diabetes had lower 25(OH)D levels than did those without diabetes (22.9 ng/mL versus 30.3 ng/mL, respectively; *p* < *0*.001). Patients with diabetes, especially those with PDR, have lower 25(OH)D levels than those without diabetes. The mean 25(OH)D levels, stratified by group, were as follows: no diabetes or ocular disease = 31.9 ng/mL; no diabetes with ocular disease = 28.8 ng/mL; no background diabetic retinopathy = 24.3 ng/mL; nonproliferative diabetic retinopathy = 23.6 ng/mL; and PDR = 21.1 ng/mL	3a
Rania NAM Reheem [168]	2013	Egypt	Cross-sectional study	200 DM patients with suspected DR (43 T1DM; 157 T2DM)	Low levels of vitamin D might be a risk marker of development or progression of diabetic retinopathy. Mean serum concentration of 1, 25 dihydroxy vitamin D 3 (1,25(OH)2D3) was significantly lower in diabetic patients with retinopathy than those with no retinopathy (NR) (51.4 ± 16.64 vs. 70.7 ± 15.56 pmol/L, *p* < 0.001). There was a significant negative correlation between the mean level of 1, 25(OH)2D3 and the degree of severity of retinopathy (*p* < 0.001). It might be advisable that detailed ophthalmologic examination is needed for diabetics whose serum 1, 25(OH)2D3 concentrations gradually decreased. The measurement of serum 1, 25(OH)2D3 concentrations could become a useful biochemical means to predict the severity of DR in patients with diabetes mellitus	3a
Hala Ahmadieh [134]	2013	Lebanon	Cross-sectional study	136 T2DM and 74 controls	Low serum 25(OH)D_3_ level was an independent predictor for diabetic neuropathy (OR 4.5 [95% CI 1.6–12]) and diabetic retinopathy (OR 2.8 [95% CI 2.1–8.0]) in patients with T2DM for vitamin D < 20 versus vitamin D ≥ 20 ng/mL after adjustment for HbA1c, age, smoking, BMI and disease duration.	4a
Myra Poon [177]	2013	Australia	Cross-sectional study	481 DM	VDD was associated with a two-fold increased risk of DR. VDD is not associated with changes in retinal vascular geometric measures, suggesting an earlier role in the time course of DR pathogenesis.	4a
Jimmy D. Alele [170]	2013	United States	Cross-sectional study	936 T2DM patients	Vitamin D status had no significant impact on the incidence of vascular events in a cohort of high-risk veterans with diabetes. No differences in the odds associated with retinopathy or renal disease onset or progression in the lowest versus highest vitamin D quartile were observed.	4b
NaokiShimo [135]	2014	Japan	Cross-sectional study	75 T1DM patients (21 with VDD; 54 without VDD)	Vitamin D deficiency was significantly associated with retinopathy in young Japanese T1DM patients. In a multivariate regression analysis, duration of diabetes (adjusted OR; 1.14, 95% CI; 1.02–1.27, *p* = 0.02) and VDD (adjusted OR; 3.45, 95% CI; 1.11–10.6, *p* = 0.03) were independent determinants of DR.	4a
R He [136]	2014	China	Cross-sectional study	625 T2DM with no DR patients, 562 T2DM with non-sight-threatening DR patients and 333 T2DM with sight-threatening DR patients	Vitamin D deficiency is an independent risk factor for diabetic retinopathy (OR 1.93) and sight-threatening diabetic retinopathy (OR 2.42) (both *p* < 0.01). The prevalence of sight-threatening diabetic retinopathy doubles when the serum 25(OH)D_3_ level is <15.57 ng/mL (odds ratio 2.38, *p* < 0.01).	4a
Sarita Bajaj [137]	2014	India	Observational caase-control study	158 T2DM patients and 130 healthy controls	Vitamin D deficiency was found to be significantly associated with neuropathy (χ2 = 5.39, df = 1, *p* = 0.020), retinopathy (χ2 = 6.6, df = 1, *p* = 0.010), and nephropathy (χ2 = 10. 52, df = 1, *p* = 0.001).	2a
Donghyun Jee [169]	2014	South Korea	Cross-sectional study	18,363 subjects	There was an inverse relationships of blood 25-hydroxyvitamin D levels with any DR and proliferative DR but only in men. After adjusting for potential confounders, including age, sex, diabetes duration, hemoglobin A1c levels, and hypertension, the odds ratios (OR) for any DR and proliferative DR among men decreased significantly in the highest blood 25-hydroxyvitamin D level quintile relative to the lowest quintile (OR, 0.37; 95% confidence interval [CI], 0.18–0.76; *p* for trend =0.004 and OR, 0.15; 95% CI, 0.03–0.83; *p* for trend = 0.043).	4a
Giacomo Zoppini [138]	2015	Italy	Cross-sectional study	715 T2DM patients	There is an inverse and independent relationship between circulating 25(OH)D_3_ levels and the prevalence of microvascular complications in patients with T2DM. Serum 25(OH)D levels decreased significantly in relation to the severity of either retinopathy or nephropathy or both. In multivariate logistic regression analysis, lower 25(OH)D levels were independently associated with the presence of microvascular complications (considered as a composite end point; OR 0.758; 95% CI 0.607 to 0.947, *p* = 0.015).	4a
Markus Herrmann [139]	2015	Australia, New Zealand, and Finland	Multinational, double-blind, placebo-controlled trial	9795 T2DM patients	Low blood 25(OH)D_3_ concentrations are associated with an increased risk of macrovascular and microvascular disease events in T2DM. A 50 nmol/L difference in blood 25OH-D concentration was associated with a 23% (*p* = 0.007) change in risk of macrovascular complications.	4a
G Bhanuprakash Reddy [167]	2015	India	Cross-sectional case-control study	82 T2DM with DR patients and 99 healthy controls	There is a possible association between vitamin D deficiency and type 2 diabetes, but not specifically with retinopathy.	3a
Celil Alper Usluogullari [140]	2015	Turkey	Cross-sectional study	557 T2DM patients (299 DPN, 223 DN and 235 DR) and 112 healthy controls	Vitamin D deficiency is associated with microvascular complications in DM patients. After adjustment, the 25 (OH) D level was confirmed to be an independent protective factor for DPN (odds ratio [OR]: 0.968, *p* = 0.004]) and DN (OR: 0.962, *p* = 0.006). The prevalence of DPN and DN increased significantly as the serum 25 (OH) D levels decreased.	4a
Nuria Alcubierre [154]	2015	Spain	Case-Control study	139 DR patients and 144 NDR patients	Patients with more advanced stages of retinopathy (grades 2–4) had lower concentrations of 25(OH)D and were more frequently vitamin D deficient as compared with patients not carrying this eye complication. The multivariate analyses showed that there was a significant association of retinopathy and 25(OH)D, even when considering other variables associated with this variable which were consistent when analyzing both the concentrations of 25(OH)D and the presence of vitamin D deficiency, as defined by a 25(OH)D <15 ng/mL (values 0.04 and 0.009, respectively.)	2a
Shokoufeh Bonakdaran [174]	2015	Iran	Cross-sectional study	235 T2DM patients (153 NDR, 64 NPDR and 18 PDR)	This study did not find any association between diabetic retinopathy and its severity and vitamin D insufficiency. Vitamin D insufficiency is not related to risk factors of diabetic retinopathy. 25(OH)D level was not significant different between NDR, NPDR and PDR groups. Correlation between 25 (OH) D level and other known risk factors of diabetic retinopathy was not significant.	3b
Lian Engelen [155]	2015	16 European countries	Prospective cohort study	532 T1DM patients	In individuals with type 1 diabetes, both higher 25(OH)D2 and 25(OH)D3 are associated with a lower prevalence of macroalbuminuria, but not of retinopathy and CVD.	3b
Adem Gungor [156]	2015	Turkey	Prospective study	50 VDD with DR patients and 50 VDD without DR patients	The results indicate that vitamin D functions as a neuroprotective component for optic nerves. Low serum 25(OH)D concentrations contribute to RNLF thinning in early-stage DR patients with VDD. The mean RNFL thickness of group 1 was significantly reduced compared to that of group 2 (*p* < 0.001). A significant relationship between the mean RNFL thickness and serum 25(OH)D concentrations was observed in group 1 (*p* < 0.001).	3a
Amy E Millen [178]	2016	United States	Population-based prospective study	1305 DM patients	25(OH)D_3_ concentrations ≥75 nmol/L were associated with lower odds of any retinopathy assessed 3 years later. ORs (95 % CIs) for retinopathy, adjusted for race and duration, were 0.77 (0.45–1.32), 0.64 (0.37–1.10), and 0.39 (0.20–0.75), *p* for trend = 0.001, for participants with 25(OH)D of 30–<50, 50–<75, and ≥75 nmol/L, respectively. Further adjustment for hypertension minimally influenced results (data not show), but adjustment for HBA1c attenuated the OR among those with 25(OH)D ≥75 (0.47 [0.23–0.96], *p* for trend = 0.030).	3a
Uazman Alam [171]	2016	UK	Prospective observational follow-up study	657 DM patients (257 NDR, 243 BDR, 135 PPDR and 22 PDR)	This study has found no association between serum 25(OH)D and the presence and severity of diabetic retinopathy or maculopathy. There was no correlation of DR with 25(OH)D (OR 1.00 (95% CI 0.98–1.02), = NS), gender, or ethnicity.	3b
Chan-Hee Jung [179]	2016	South Korea	Cross-sectional study	257 T2DM patients (men: 21 deficient, 60 insufficient and 30 sufficient; women: 63 deficient, 72 insufficient and 11 sufficient)	Serum 25(OH)D level < 10 ng/mL is independently associated with increased DPN in male patients and increased DN in female patients with T2DM. For men, the prevalence of DPN was significantly higher in vitamin D deficient patients than those with insufficient or sufficient vitamin D (38%, 11.7%, and 10%, respectively; *p* = 0.005). The prevalence of DR and DN was not different according to the vitamin status. For women, the prevalence of DN was significantly higher in patients with vitamin D deficiency than in the other 2 vitamin D status (40%, 20.6%, and 0%, for vitamin D-deficient, vitamin D-insufficient, and vitamin D-sufficient groups, respectively; *p* = 0.007) and the prevalence of DPN and DR was not different among 3 status.	4a
Xianglong Yi [180]	2016	China	Case-control study	22 PDR patients, 29 NPDR patients and 24 healthy controls	There is a potential protective effect of 1,25(OH)2 D3 in DR, whereas supplementation with 1,25(OH)2 D3 might be an effective strategy for preventing the development of DR	2a
M Long [141]	2017	United States	Retrospective cross-sectional study	842 DR patients, 301 mild NPR patients, 195 severe NPR patients and 106 PDR	Vitamin D deficiency associated with severe DR in patients with well controlled glycasemia. After adjusting gender, ethnicity and duration of diabetes, the interaction of HbA1 with vitamin D deficiency significantly affected retinopathy severity (*p* = 0.029).	4b
Banu Boyuk [157]	2017	Turkey	Cross-sectional case-control study	206 T2DM patients and 34 healthy controls	There is an inverse relationship between the circulating 25(OH) D level and the prevalence of retinopathy and neuropathy in T2DM patients. The level of serum 25-hydroxyvitamin D (25(OH) D) was significantly lower in the T2DM patients (11.16 ± 3.99 ng/mL vs. 15.58 ± 3.16 ng/mL; *p* < 0.05). Serum 25(OH) D levels weresignificantly lower in the presence of retinopathy and neuropathy (*p* < 0.05 for both), but no significant association between Vitamin D level and microalbuminuria was found.	3b
Anurag Chaurasia [158]	2017	India	Case-control study	120 DM patients (40 No BDR, 42 mild-mod NPDR and 38 sev–v.serv NPDR and PDR) and 100 NDM patients (58 no DM and ocular diseases and 42 no DM with ocular diseases)	Diabetic subjects, especially those with NPDR (severe and very severe grade) and PDR have lower Vitamin D levels than those without diabetes. Subjects with vitamin D insufficiency are at risk of having retinopathy 9.75 times that of vitamin D sufficient subjects (Odds Ratio 9.75; 95% C.I. 1.96 to 48.46). The mean vitamin D level in Diabetics (20.7 ± 6.91 ng/mL) is significantly less than that in non-diabetics (27.51 ± 5.53 ng/mL) (*p* < 0.0001). Also, diabetics have significantly higher proportion of subjects with vitamin D insufficiency (91.3%) as compared to Non-Diabetics (68%).	4a
Beteal Ashinne [142]	2018	India	Retrospective study	3054 T2DM patients	Lower serum 25(OH)D_3_ was associated with increased severity of DR and the presence of vitamin D deficiency was associated with a two-fold increased risk for PDR. A statistically significant difference in the serum vitamin D means of these categorizations: no DR (13.7 ± 2.1 ng/mL), non-sight threatening DR (12.8 ± 2.1 ng/mL), sight threatening DR (11.1 ± 2.2 ng/mL), (*p* < 0.001).	4a
Abdulbari Bener [143]	2018	Turkey	Cross-sectional study	638 DM patients	Vitamin D deficiency is considered as a risk factor for DR and hearing loss among diabetic patients.	
Krishnamoorthy Ezhilarasi [181]	2018	India	Case-control study	200 T2DM patients, 216 T2DM with microvascular complications, 198 T2DM with macrovascular complications and 252 controls with nomal glucose tolerance	VDR (rs1544410) SNP was found to be associated with decreased serum (25[OH]D) levels in both micro-macrovascular complications of T2DM among South Indian Population.	4a
Josef Richter [159]	2018	Czech Republic	Clinic Controlled Trial	52 DR patients (Vitamin D and Beta-glucan supplement, Vitamin D and placebo supplement and vitamin D supplement only)	Significant vitamin D deficits were found in all cases, even after three months of supplementation with vitamin D.	7/9 * (RoB)
Martina Závorková [182]	2018	Czech Republic	Clinic Controlled Trial	54 DR patients (beta-glucan and vitamin D supplement, vitamin D and placebo supplement, vitamin D supplement only, no supplementation	Glucan and vitamin D supplementation strongly influence lipid metabolism and have positive effects on human health.	7/9 * (RoB)
Gauhar Nadri [144]	2019	India	Cross-sectional study	24 DM without DR patients, 24 DM with NPDR patients and 24 DM with PDR patients	Serum vitamin D levels of ≤ 18.6 ng/mL serve as sensitive and specific indicator for proliferative disease, among patients of DR. Univariate ordinal logistic regression analysis found vitamin D as a significant predictor of severity of diabetic retinopathy {OR (95% CI) = 1.11 (1.06–1.16) (*p* < 0.01 or *p* < 0.001)}. ROC curve analysis demonstrated vitamin D cut off value of 18.6 ng/mL to be significantly associated with NPDR and PDR	4a
Jing Yuan [145]	2019	China	Cross-sectional study	889 T2DM patients	Vitamin D deficiency is significantly associated with risk of PDR. The odd ratio in VDD individuals was significantly increased (1.84, 95% CI 1.18–2.86) for DR, 1.60 (95% CI 1.06–2.42) for PDR, compared with those in vitamin D sufficient individuals, adjusted by age, sex, blood pressure, kidney function, diabetic duration, and HbA1c.	4a
Heng Wan [146]	2019	China	Cross-sectional study	4767 DM patients	Lower serum 25(OH)D_3_ concentration is significantly associated with higher prevalence of DR in middle-aged and elderly diabetic adults. Compared with the first 25(OH)D quartile (relatively lower concentraion), participants in the fourth quartile (relatively higher concentration) had a lower prevalence of high ACR (OR 0.77, 95% CI 0.61, 0.96) (*p* for trend <0.01). For DR, the OR of DR for the fourth 25(OH)D quartile when compared with the first quartile was 0.62 (95% CI: 0.47, 0.82; *p* for trend <0.01).	4a
Abdulhalim Senyigit [160]	2019	Turkey	Cross-sectional study	163 T2DM patients and 40 controls	Low serum 25-OHD levels were found to be associated with the development of diabetes and complications. Serum 25(OH)D levels in all patients were significantly lower than the control group (*p* < 0.05). Levels of 25(OH)D for those with complications were lower than that without complications. (*p* values for nephropahty and retinopathy groups were <0.001 while that for neuropahty was <0.01). Low serum 25-OHD levels may be a consequence of even worse metabolic control of diabetes.	3a
Abdulla Almoosa [161]	2019	Bahrain	Prospective observational study	300 T2DM patients (150 NDR, 99 NPDR and 51 PDR)	VDD was commonly found in type II DM patients. Lower serum 25(OH)D levels were associated with more severe DR.	3b
Mehrdad Afarid [148]	2020	Iran	Cross-sectional study	21 DM with NPR patients, 9 DM with PDR patients and 30 DM with no DR patients	Patients with DR had lower levels of serum vitamin D compared with those without retinopathy, especially those with severe NPDR and PDR	3a
Amy E. Millen [178]	2020	Portugal	Retrospective observational study	182 T1DM patients	Lower levels of vitamin D were associated with an increased prevalence of diabetic retinopathy in patients with T1DM, after adjusting for possible confounders. The lower circulating 25(OH)D levels were significantly associated with greater prevalence of DR after adjusting for confounders (OR = 0.94; 95% CI 0.90–0.99, *p* = 0.023).	4a
Lina H. M. Ahmed [150]	2020	Qatar	Case-control study	274 T2DM patients and 222 healthy controls	Vitamin D_3_ was associated with diabetic retinopathy whilst vitamin D_2_ was not.	2a
Lina H. M. Ahmed [162]	2021	Qatar	Cross-sectional study	460 T2DM patients and 290 healthy controls	Vitamin D_3_ metabolites were associated with diabetic retinopathy, whereas total vitamin D levels were not, suggesting that endogenous vitamin D(3) metabolites are a better measure of diabetic microvascular complications. The lower 1,25(OH)2D3 levels were associated with diabetic retinopathy (*p* = 0.006) hypertension and dyslipidemia (both *p* = 0.01) and CAD (*p* = 0.012); while no association between either total 1,25(OH)2D or 1,25(OH)2D3 levels with diabetic neuropathy, PAD or CAD.	3a
Alexandra E. Butler [147]	2020	Qatar	Cross-sectional study	460 T2DM patients and 290 healthy controls	Lower 25(OH)D(3) was associated with retinopathy (*p* < 0.03) and dyslipidemia (*p* < 0.04), but not neuropathy or vascular complications	3a
Ying Xiao [166]	2020	China	Cross-sectional study	4284 T2DM patients	In unadjusted analyses, DR was associated with VDD status (PR: 1.147; 95% CI: 1.025–1.283), the associate retained after adjusted with age and sex and other demographic and physical measurements. However, the significance diminished after adjusting all confounders (PR: 1.093; 95% CI: 0.983–1.215).	4a
Gauhar Nadri [163]	2021	India	Cross-sectional study	66 T2DM patients (22 No DR, 22 NPDR and 22 PDR) and 22 controls	Low serum vitamin D levels correlate with increased severity of DR.	4a
Li Lu [151]	2021	China	Retrospective study	55 PDR patients, 25 non-diabetic patients with idiopathic macular hole patients and 10 NDR patients	In ROC-curve analyses, both serum and vitreous 25(OH)D showed discriminatory ability in predicting DR (NPDR and PDR) and PDR. In DR prediction, they obtained the same area under curve (AUC) of 0.77. Serum 25 (OH) D has a better predictive value (AUC: 0.77) than serum 25 (OH) D (AUC: 0.66) in PDR prediction.	4a
Wei-Jing Zhao [152]	2021	China	Cross-sectional study	815 T2DM patients	Vitamin D deficiency is independently associated with higher risk of diabetic peripheral neuropathy and DR, but not diabetic retinopathy, in T2DM patients. Univariate analysis showed that the 25 (OH) D was significantly correlated with DPN (odds ratio [OR]: 0.969, 95% confidence interval [CI]: 0.950–0.989, *p* = 0.003) and DN (OR: 0.950, 95% CI: 0.928–0.973, *p* < 0.001), but not with DR (OR: 1.014, 95% CI: 0.994–1.034, *p* = 0.165). Multiple logistic regression analysis after adjustment showed that the 25 (OH) D level was an independent protective factor for DPN and DN.	4a
Xin Zhao [153]	2021	China	Retrospective study	636 T2DM patients (466 NDR, 120 BDR and 50 PDR)	A close association was observed between 25(OH)D_3_ level and DR in the elderly male patients and postmenopausal women with T2DM. There was a significant difference was observed among the three groups in men and women (men: χ2 = 7:75, *p* < 0.05; women: χ2 = 7:75, *p* < 0.05)	4a
Mehmet Balbaba [172]	2021	Turkey	Prospective study	20 T2DM-DR patients, 20 T2DM-NDR patients and 20 healthy controls	Vitamin D levels were similar between diabetic patients with and without DR and healthy control subjects	3b
Saeed Karimi [183]	2021	Iran	Prospective comparative case series study	71 diabetic macular edema patients	In diabetic macular edema patients with vitamin D deficiency, vitamin D supplement therapy had some beneficial effects on central macular thickness reduction following three injections of intravitreal bevacizumab; nevertheless, these effects were not statistically significant	8/9 * (RoB)
Elise Girard [173]	2021	French Guiana	Cross-sectional study	361 DM patients	There was no significant difference between type of diabetes and VD deficiency. There was no significant relation between nephropathy and vitamin D deficiency even after adjustment with confounders. Patients with diabetic retinopathy had significantly greater median vitamin D concentrations than those without retinopathy (respectively, 31 ng/mL (interquartile range (IQR) = 23–34) vs. 26 ng/mL (IQR = 23–32), *p* = 0.03).	4a
José M. Castillo-Otí [164]	2021	Spain	Case-Control study	385 T2DM patients (30 with DR, 335 without DR)	Levels of 25(OH)D and treatment of diabetes were significantly associated with DR after adjusting for other risk factors. Patients with both 25(OH)D and 1,25(OH)2D less than or equal to 16 ng/mL and 29 pg/mL respectively had a greated risk of DR (OR 5.21, 95% CI: 1.76, 15.42; *p* = 0.003).	4a
Martina Tomić [165]	2021	Croatia	Cross-sectional study	94 T2DM patients (69 without DR and 25 with NPDR)	Hypovitaminosis D is often in T2DM, especially in those with proliferative DR.	3b

Diabetic retinopathy (DR), background diabetic retinopathy (BDR), non-proliferative retinopathy (NPR), preproliferative diabetic retinopathy (pre-PDR), proliferative diabetic retinopathy (PDR), Diabetes mellitus (DM), Type 1 diabetes mellitus (T1DM), type 2 diabetes mellitus (T2DM), diabetic kidney disease (DKD); * The Effective Practice and Organisation of Care (EPOC) RoB Tool for randomized trials; # LEGEND for case-control, cohort, and cross-sectional studies, rating of the studies follow the guidelines from LEGEND.

**Table 5:** Summary of studies related to dry eye syndrome included.

First Author	Years	Country	Study-Design	Sample Size	Main Finding	Rate #
B Bang [211]	1999	Denmark	Case-control study	41 Primary SS patients and 596 healthy controls	Vitamin D metabolism may be involved in the pathogenesis of primary SS.	7/9 * (RoB)
Anat Galor [212]	2014	United States	Cross-sectional study	247 subjects	Higher vitamin D levels had a favorable but small effect on DES symptoms. Higher vitamin D levels were significantly associated with lower DES symptoms (−1.24 decrease for every 10-U increase in vitamin D, *p* = 0.01).	2b
B E Kurtul [213]	2015	Turkey	Case-control study	34 Vitamin D deficiency patients and 21 healthy controls	Vitamin D deficiency decreases the TBUT and Schirmer test values and may be associated with dry-eye symptoms in non-SS. The TBUT scores and Schirmer-1 test values of study group were significantly lower than that of the control group (*p* = 0.01 and 0.007, respectively).	3a
Pelin Yildirim [214]	2015	Turkey	Case-control study	50 Premenopausal women with vitamin D deficiency and 48 healthy controls	Dry eye and impaired tear function in patients with vitamin D deficiency may indicate a protective role of vitamin D in the development of dry eye. There were significant differences between the vitamin D deficiency group and control group for the frequencies of the patients with dry eye (*p*-value for the results of Schirmer’s test, TBUT and OSDI were 0.001, 0.001 and 0.003 respectively).	3a
Donghyun Jee [227]	2016	South Korea	Cross-sectional study	1679 DES patients and 14,717 No DES patients	The present study does not support an association between serum 25-hydroxyvitamin D levels and DES.	2a
Ki Won Jin [215]	2016	South Korea	Retrospective observational study	79 DES patients	TBUT and secretion were correlated with serum vitamin D levels. Tear break-up time (TBUT) and tear secretion were shorter in the vitamin D-deficient group compared to the sufficient group (*p* = 0.022 and *p* = 0.004). Vitamin D status may be an important factor for dry eye syndrome.	4a
Sam Young Yoon [216]	2016	South Korea	Cross-sectional study	1822 DES patients and 15,720 healthy controls	Low serum 25(OH)D_3_ levels and inadequate sunlight exposure are associated with DES in Korean adults. Inadequate sunlight exposure time (odds ratio [OR], 1.554; 95% confidence interval [CI], 1.307–1.848), low serum 25(OH)D level (OR, 1.158; 95% CI, 1.026–1.308) and indoor occupation (OR, 1.578; 95% CI, 1.389–1.814) were the risk factors for DES. Sufficient sunlight exposure or vitamin D supplementation may be useful in DES treatment.	8/9 * (RoB)
Rohit Shetty [217]	2016	India	Cross-sectional study	52 evaporative dry eye patients and 43 healthy controls	In the evaporative dry eye cohort, there were strong inverse correlation between the vitamin D levels and OSDI scores (total and discomfort- and vision-related subscales) but not total corneal DC density, DCs without dendritic process, or subbasal nerve plexus features.	2b
Rohit Shetty [218]	2016	India	Cross-sectional study	19 Mild dry eye signs with exaggerated symptoms patients and 19 healthy controls	Decreased serum vitamin D was associated with exaggerated symptoms in dry eye patients with mild dry eye signs. An inverse correlation (r = −0.569; *p* = 0.0110) was observed between serum vitamin D levels and OSDI score in the patient cohort. Tukey’s multiple comparisons test showed a significant difference between the OSDI score of patients with serum vitamin D less than or equal to 10 ng/mL and greater than 20 ng/mL.	7/9 * (RoB)
Seok Hyun Bae [219]	2016	South Korea	Retrospective observational study	105 DES refractory to conventional treatment and vitamin D deficiency patients	Vitamin D supplementation is effective and useful in the treatment of patients with DES refractory to conventional treatment and with vitamin D deficiency. The TBUT in males was increased after 2 weeks compared to pre–treatment and in female it was increased after 2 and 6 weeks compared to pre-treatment (*p* = 0.041, <0.001 and <0.001, respectively, paired *t*-test). OSDI score in men was lower at 6 weeks compared to pre-treatment and in women it was lower at 10 weeks compared to pre-treatment (*p* = 0.033 and 0.012, respectively, paired *t*-test).	3a
Min Ji Kim [228]	2017	South Korea	Cross-sectional study	1428 DES patients and 7921 healthy controls	Severe vitamin D deficiency was associated with dry eye in an unadjusted model (*p* = 0.01), but the association was not statistically significant after adjustment (*p* = 0.49, Vit D insufficiency; *p* = 0.33, VDD; *p* = 0.18, sever VDD). OR was 1.24 (95% CI, 0.66 to 2.41) for vitamin D insufficiency, 1.30 (95% CI, 0.75 to 2.25) for vitamin D deficiency, and 1.42 (95% CI, 0.83 to 2.41) for severe vitamin D deficiency after adjusting confounders.	4a
Da-Hye Jeon [229]	2017	South Korea	Cross-sectional study	393 DES patients and 347 healthy controls	Serum vitamin D levels are not associated with DES after adjusting confounders. Higher serum vitamin D levels were associated with a non-significantly reduced risk of DED in the crude analysis (odds ratio [OR], 0.991; 95% confidence interval [CI], 0.971 to 1.011) and in the adjusted analysis (OR, 0.988; 95% CI, 0.966 to 1.010).	8/9 * (RoB)
Yi-Fang Meng [220]	2017	China	Case-control study	70 DES patients and 70 healthy controls	A significant association between serum 25(OH)D level and DES incidence was detected. In Pearson correlation analysis, serum 25(OH)D level was associated with increased Schimer test I (r = 0.8248, *p* < 0.001). In addition, there was an inverse correlation between serum 25(OH)D and ODSI scores (r = −0.3348, *p* = 0.005) and TBUT (r = −0.6806, *p* < 0.001).	4a
Reiko Arita [231]	2017	Japan	Clinical trial	8 MGD patients and 6 healthy controls	Topical eyelid application of an analog of the active form of vitamin D3 was found to be safe as well as to improve the condition of patients with obstructive meibomian gland dysfunction.	7/9 * (RoB)
Muhammed Kizilgul [232]	2017	United States	Clinical trial	44 DES patients	As a consequence of the presence of VDR and 1α-hydroxylase in different parts of the eye, vitamin D replacement improves tear hyperosmolarity that is considered to be induced by ocular surface inflammation. The change of TFO was negatively correlated with the change of 25(OH)D3 before and after replacement in patients with dry eye disease (r = −0.390, *p* = 0.049).	2b
Chih-Huang Yang [233]	2017	Australia	Case-control study	29 DES patients and 29 healthy controls	Low vitamin D levels (<50 nmol/l) were associated with dry eye symptoms in older individuals but not those diagnosed with dry eye. Vitamin D supplement increased the vitamin D levels, and improved dry eye symptoms, the tear quality and ocular surface conditions.	3a
Goktug Demirci [221]	2018	Turkey	Cross-sectional observational study	30 Vitamin D deficiency patients and 30 healthy controls	Vitamin D deficiency is associated with tear hyperosmolarity and tear film dysfunction. The Schirmer I test values and TBUT measurements for VDD were significantly lower compared with controls (*p* < 0.001). Patients with vitamin D deficiency may be prone to dry eye.	3a
Pooja Khamar [222]	2019	India	Cross-sectional study	47 evaporative dry eye patients and 33 healthy controls	Significantly lower vitamin D was observed in DED patients (*p* < 0.05). These dysregulated tear factors showed significant associations with DED signs and symptoms.	2a
Hwang Jin Sun [234]	2019	South Korea	Case-control study	116 DED patients (52 VDD and 64 non-VDD)	The OSDI score was decreased in the IM group (intramascular supplementation of vitamin D for 2 weeks) after cholecalciferol supplementation compared with pretreatment, whereas that in the none group and oral group was not different between after cholecalciferol supplementation and pretreatment. The effect of topical carbomer-based lipid–containing artificial tears and hyaluronate was dependent on serum 25HD levels.	4a
Jee Hye Lee [223]	2020	South Korea	Retrospective study	74 Primary SS patients	Serum 25(OH)D_3_ level might be associated with dry eye severity in primary SS. Among dry eye parameters, the corneal staining score, conjunctival staining score, Schirmer I value, and TBUT were statistically worse in the serum 25(OH)D3 deficiency group compared with the normal group (*p* < 0.05). The conjunctival staining score showed a significant difference between the deficiency group and insufficiency group (*p* < 0.05).	8/9 * (RoB)
Palak Watts [235]	2020	India	Prospective study	90 DED with deficient serum 25(OH)D_3_ levels patients	Vitamin D supplementation leads to earlier and significant improvement in TBUT, Schirmer’s, and OSDI score in patients with vitamin D deficient DED.	2b
Seyhan Dikci [226]	2020	Turkey	Case-control study	36 Vitamin D deficiency patients and 27 healthy controls	Vitamin D deficiency may lead to dry eye causing conjunctival squamous metaplasia and loss of goblet cells on the ocular surface. Serum vitamin D levels had moderate negative correlation with CIC results (r = −0.595; *p* < 0.001), and mild positive correlation with TBUT scores (r = 0.384, *p* = 0.002). There was no correlation between serum vitamin D levels and Schirmer II test and OSDI scores (r = 0.169, *p* = 0.185, r = 0.163, *p* = 0.202, respectively). No correlation was found between age and Schirmer’s II, TBUT, OSDI scores, and CIC results.	7/9 * (RoB)
Emine Esra Karaca [236]	2020	Turkey	Clinical controlled trial	40 Vitamin D deficiency patients	Vitamin D replacement appears to improve ocular surface in individuals with vitamin D deficiency.	3a
Aksoy Aydemir, Gozde [225]	2021	Turkey	Prospective cross-sectional study	90 Pediatric patients with type 1 diabetes mellitus patients and 80 healthy controls	The tear measurements of the pediatric type 1 diabetes mellitus were lower than those in the healthy pediatric control group. The accompanying VDD made this situation more pronounced. The correlations between the vitamin D level and the Schirmer test, OSDI score, CSS, TF–BUT measurements, TMA, and TMH values were examined in T1DM group and control groups. Although there was a significant correlation in all measurements in T1DM group, there was no correlation in control groups.	4a
Shima Fukuoka [224]	2021	Japan	Cross-sectional study	300 subjects	High intake of total fat, SFAs, oleic acid, and vitamin D may be inversely associated with the prevalence of MGD in Japanese individuals. Vitamin D intake in the MGD group was significantly lower than that in the non-MGD group (*p* = 0.039). Multivariate adjusted odds ratios (95% confidence intervals) between extreme quintiles of intake of vitamin D for MGD prevalence was 0.38 (0.17–0.87).	8/9 * (RoB)
Alireza Eslampoor [237]	2022	Iran	RCT	100 dry eye disease patients with concurrent vitamin D deficiency	Vitamin D supplementation as an adjuvant to routine dry eye treatment improves ocular surface hemostasis parameters, results in better tear stability and a more improved tear osmolarity in patients with vitamin D deficiency.	4a
Jain Nikita [238]	2022	India	Hospital-based cross-sectional	60 VDD patients and 60 normal levels subjects	A significant difference in the mean values of Schirmer I and Schirmer I test (with anesthesia) (*p* < 0.001) was seen between the case and control groups. A significant difference in the mean values of TFBUT (*p* < 0.001) and OSDI scores (*p* < 0.01) was also seen between the two groups.	7/9 * (RoB)

Sjögren’s syndrome (SS), Dry eye syndrome (DES), Tear film break-up time (TBUT), Ocular surface disease index (OSDI); * The Effective Practice and Organisation of Care (EPOC) RoB Tool for randomized trials; # LEGEND for case-control, cohort, and cross-sectional studies, rating of the studies follow the guidelines from LEGEND.

**Table 6:** Summary of studies related to thyroid eye disease included.

First Author	Years	Country	Study-Design	Sample Size	Main Finding	Rate #
Ama Sadaka [245]	2019	United States	Retrospective study	35 TED patients	20% and 31% prevalence of vitamin D deficiency and insufficiency were found in TED, respectively.	4a
Curtis J Heisel [246]	2020	United States	Retrospective case-control study	89 TED patients (TED), 89 GD patients without TED (GD), and 2 healthy control groups matched 4:1 to the cases; 356 healthy control patients matched to the TED group (HC TED), and 356 HC patients matched to the GD (HC GD)	Low serum vitamin D is associated with TED diagnosis. Assessing and supplementing vitamin D levels may be an important addition to the early management of GD patients.	4a
Tereza Planck [247]	2018	Sweden	Cross sectional study	Epidemiological part—292 GD patients and 2305 healthy controls Clinical part—219 GD patients Relapse analysis after antithyroid drug treatment part —100 GD patients Genetic part—708 GD patients with (*n* = 245) or without (*n* = 459) ophthalmopathy and 1178 sex-matched controls	Patients with GD had lower vitamin D levels compared to the general population; however, the vitamin D levels did not affect the laboratory or clinical parameters of GD. SNPs in the VDR influenced the risk of GD through mechanisms other than reducing the vitamin D levels.	4a

Thyroid eye disease (TED), Graves’ disease (GD); # LEGEND for case-control, cohort, and cross-sectional studies, rating of the studies follow the guidelines from LEGEND.

**Table 7:** Summary of studies related to uveitis included.

First Author	Years	Country	Study-Design	Sample Size	Main Finding	Rate #
Stephanie M. Llop [251]	2018	United States	Retrospective case-control study	333 Uveitis patients, 103 scleritis patients and 329 controls	Hypovitaminosis D was associated with increased risk of ocular inflammation	4a
Lindsay A Grotting [255]	2017	United States	Retrospective case-control study	100 Noninfectious anterior uveitis patients and 100 healthy controls	Lower vitamin D levels are associated with an increased risk of noninfectious anterior uveitis.	4a
Xianglong Yi [256]	2011	China	Case-control study	8 active VKH patients, 7 inactive VKH patients and 8 healthy controls	These findings suggest that decreased expression of 1,25(OH)_2_D_3_ may be involved in the development of VKH disease. 1,25(OH)_2_D_3_ may be potentially used in the treatment of this disease.	4b
Zeynep Dadaci [254]	2016	Turkey	Case-control study	20 acute anterior uveitis patients and 20 healthy controls	Significantly low serum levels of vitamin D was found in patients with acute anterior uveitis, which suggest that vitamin D deficiency may play a role in the pathogenesis of anterior uveitis.	4a
TC Mitulescu [253]	2016	Romania	Case-control study	11 AS with AAU patients, 23 AS patients without AAU patients and 18 healthy controls	Altered levels of Vit D affect the balance between LL-37, IL-8 and Serum Amyloid A, suggesting an association with AAU, an extra-articular manifestation of AS.	4b
Lucia Sobrin [252]	2018	United States	Retrospective case-control study	558 noninfectious uveitis patients and 2790 healthy controls	Hypovitaminosis D may be a risk factor for noninfectious uveitis.	4a
Zelia K. Chiu [259]	2020	Australia	Prospective case-control study	74 active and 77 inactive noninfectious uveitis patients and 594 local general population controls	Participants with active uveitis showed significantly lower serum 25(OH)D_3_ levels than inactive uveitis patients and local population-based estimates. Vitamin D supplementation was found to be associated with decreased uveitis activity, as was sun exposure in those with vitamin D deficiency.	4a
Ma’an Abdullah Al-Barry [25]	2016	Arabia	Cross-sectional study	39 VKH patients and 50 healthy controls	low vitamin D levels might play a role in VKH pathogenesis and mutations in genes involved in vitamin D anabolism and catabolism might be of importance in VKH pathobiology.	3b
Julien Rohmer [257]	2020	France	Retrospective study	59 Uveitis patients	The measurement of serum 25(OH)D_3_ and 1,25(OH)_2_D_3_ levels is a useful tool in the etiological workup of patients with unexplained uveitis, since a high 1,25(OH)_2_D_3_/25(OH)D_3_ ratio is suggestive of ocular sarcoidosis.	4a
Claudia Sengler [258]	2018	Germany	Prospective observational, controlled multicenter study	360 juvenile idiopathic arthritis (JIA) patients and 360 healthy controls	25(OH)D_3_ deficiency was common and associated with higher disease activity and risk of developing JIA-associated uveitis.	3a
Marta Mora Gonzalez [250]	2018	United States	Cross-sectional population-based study	25 uveitis patients and unknown number of non-uveitis controls	None of the 25 patients were found to have serum vitamin D values indicative of deficiency (less than or equal to 30 nmol/L).	3b

Vogt-Koyanagi-Harada (VKH), ankylosing spondylitis (AS), acute anterior uveitis (AAU); # LEGEND for case-control, cohort, and cross-sectional studies, rating of the studies follow the guidelines from LEGEND.

**Table 8:** Summary of studies related to tumor included.

First Author	Years	Country	Study-Design	Sample Size	Main Finding	Rate #
Fabiola Mejía-Rodríguez [266]	2021	Mexico	Case-control study	126 Sporadic RB patients and 102 healthy controls	Latitude and the number of days exposed to the spring–summer season during 6 to 11.9 months of life were negatively associated with sporadic retinoblastoma in children who had exclusive maternal lactation.	4a
Manuela Orjuela-Grimm [267]	2021	Mexico	Case-control study	259 Unilateral RB patients, 120 bilateral RB and 132 healthy controls	Sun exposure in early childhood is protective for retinoblastoma and may decrease degree of intraocular spread in children with bilateral RB.	4a
Schundeln Michael M [268]	2015	Germany	Cross-sectional study	14 Unilateral RB patients and 19 bilateral RB	51.7% of RB patients and 13.7% of RB patients were vitamin D deficient and severe vitamin D deficient, respectively.	3b

Retinoblastoma (RB); # LEGEND for case-control, cohort, and cross-sectional studies, rating of the studies follow the guidelines from LEGEND.

**Table 9:** Summary of studies related to cataract included.

First Author	Years	Country	Study-Design	Sample Size	Main Finding	Rate #
Aidenloo Naser Samadi [280]	2021	Iran	Cross-sectional study	216 nuclear cataract patients, 336 cortical cataract patients, 140 posterior sub-capsular cataract patients, 549 mixed cataract patients and 200 normal controls	Serum 25(OH)D_3_ levels were inversely associated with the risk of nuclear cataract and cortical cataract and not associated with posterior sub-capsular cataract or mixed cataract in women. No association between vitamin D and cataractogenesis in men.	4a
Donghyun Jee [281]	2015	South Korea	Population-based cross-sectional study	18,804 participants (7993 age-related cataracts, 1332 with a history of cataract surgery and 9479 no cataract participants)	Men with higher serum 25(OH)D_3_ levels have a lower risk for the age-related cataract; while no association were found in women.	4a
Sangshin Park [274]	2016	South Korea	Cross-sectional study	16,086 participants (cortical, 1562; nuclear, 4116; anterior subcapsular, 174; posterior subcapsular, 87; mixed, 1013; Q1, 3215; Q2, 3214; Q3, 3217; Q4, 3226; and Q5, 3214)	Serum 25(OH)D_3_ levels were inversely associated with the risk of nuclear cataract.	4a
Caglar Okem [278]	2021	Turkey	Sectional case-control study	37 cataract patients and 53 healthy controls	Vitamin D deficiency as associated with early age-related cataract.	4a
Prethy Rao [280,282]	2015	United States	Cross-sectional study	1278 participants (nuclear cataract, 516; Q1, 256; Q2, 257; Q3, 254; Q4, 256; and Q5, 255)	No significant association between serum 25(OH)D_3_ levels and nuclear cataract.	4a
Craig J Brown [279]	2015	United States	Cross-sectional	175 cataract patients	Vitamin D deficiency was associated with posterior subcapsular cataract.	4b
K Atalay [277]	2020	Turkey	Hospital-based cross-sectional study	26 posterior subcapsular cataract patients and 53 age-related cataract without posterior subcapsular cataract patients	Patients with age-related cataract without posterior subcapsular component showed statistically significantly lower vitamin D level than 20 ng/mL (vitamin D insufficiency level).	4b
Min-Chul Cho [283]	2020	South Korea	Prospective study	87 senile cataract patients and 49 diabetic cataract patients	Higher 25(OH)D_3_ levels in aqueous humor was associated with diabetic cataract.	3a
Marwa Mahmoud Abdellan [276]	2019	Egypt	Case-control study	325 cataract patients and 385 healthy controls	Vitamin D deficiency may have a role in age-related cataract patients.	4a

# LEGEND for case-control, cohort, and cross-sectional studies, rating of the studies follow the guidelines from LEGEND.

## Data Availability

Not applicable.
